# A pectin acetyl‐transferase facilitates secondary plasmodesmata formation and RNA silencing movement between plant cells

**DOI:** 10.1111/tpj.70194

**Published:** 2025-05-12

**Authors:** Florence Jay, Florian Brioudes, Lazar Novaković, André Imboden, Yoselin Benitez‐Alfonso, Olivier Voinnet

**Affiliations:** ^1^ Department of Biology Swiss Federal Institute of Technology (ETH‐Zürich) Zürich 8092 Switzerland; ^2^ School of Biology, Centre for Plant Sciences, and Astbury Centre University of Leeds Leeds LS2 9JT UK

**Keywords:** RNA silencing, movement, plasmodesmata, pectin, cell wall

## Abstract

Some silencing small (s)RNAs, comprising micro (mi)RNAs and small‐interfering (si)RNAs, move between plant cells to orchestrate gene expression and defense. Besides possible redundancy or embryo lethality, a prevalent challenge in genetic studies of mobile silencing is to discriminate *bona fide* alterations to sRNA movement from impaired cell‐autonomous sRNA activity within silencing‐recipient cells. Without such clarifications, cell‐to‐cell mobility factors are yet to be unequivocally identified. Consequently, known properties of sRNA movement, including contextuality and directionality, remain poorly explained. Circumstantial evidence and synthetic biology pinpoint plasmodesmata (PDs) – the pores traversing plant cell walls (CWs) – as the likely channels involved. Yet, how plants control the number of primary and secondary PDs developing respectively before and after CW formation remains largely unknown. Here, we address these intertwined issues in Arabidopsis using a forward screen for compromised epidermis‐to‐mesophyll movement of an artificial (a)miRNA. We identify a pectin acetyl‐transferase mutation that, we demonstrate, reduces amiRNA physical trafficking but also impedes siRNA, GFP, and viral movement by decreasing the frequency of leaf secondary PDs. sRNA movement at leaf interfaces involving primary PDs remains unaffected, however, as does miRNA and GFP cell‐to‐cell mobility in roots, hinting at how movement's contextuality and directionality might be achieved. We also show that reducing de‐esterified pectin depolymerization decreases leaves' symplasmic connectivity, whereas defective pectin biogenesis increases PD number. Combining genetics with antibody‐based pectin probing and atomic force microscopy helps delineate a mechanistically coherent framework whereby pectin esterification and/or abundance impact CW loosening, a process required for CW extension during which secondary PDs form to enable macromolecular trafficking.

## Significance Statement

This work identifies a role for pectin and its modifications in controlling the number of plasmodesmata formed within existing plant cell walls. The underlying processes influence macromolecular cell‐to‐cell trafficking, including that of mobile silencing small RNAs.

## INTRODUCTION

Plant cell walls (CWs) are composed of cellulose microfibrils and cross‐linking glycans such as xyloglucans and xylans, both embedded within a pectin matrix. CWs are traversed by plasma membrane (PM)‐lined pores called plasmodesmata (PDs) that enable symplasmic transport of solutes, photoassimilates, hormones, and macromolecules (Brunkard & Zambryski, 2017). Primary and secondary PDs exist, formed respectively before and after CW formation, and are only distinguishable developmentally as opposed to structurally (Burch‐Smith et al., 2011). Both associate with PLASMODESMATA‐LOCATED PROTEINs (PDLPs), among which PDLP3 defines a core PD marker (Amari et al., 2010). Two non‐mutually exclusive mechanisms influence the extent of PD‐mediated cell–cell trafficking, with the first pertaining to permeability. Reversible redox‐regulated callose deposition around PD's necks can indeed dynamically control their aperture (Benitez‐Alfonso et al., 2009; Brunkard & Zambryski, 2017; Peters et al., 2021). This process requires the concerted actions of callose synthases and PDLPs, including PDLP5 (Saatian et al., 2023), possibly upon stimulation of certain PM‐anchored receptor‐like kinases (RLKs) (reviewed in Tee & Faulkner, 2024). Mutations in *INCREASED SIZE EXCLUSION LIMIT 3* (*ISE3*) and *ISE4* also alter symplasmic connectivity by disrupting TOR signaling, which monitors changes in sugar availability; upon glucose activation, TOR might decrease PD‐mediated transport (Brunkard et al., 2020).

PDs' permeability also evolves during their developmental maturation. In young growing leaves, so‐called “simple” primary and secondary PDs with single connections allow diffusion of macromolecules including soluble (free) GFP (Oparka et al., 1999). As leaves age, they mature into branched/funnel‐shaped “complex” PDs with reduced diffusion potential at least at certain cell–cell interfaces (Burch‐Smith et al., 2011; Oparka et al., 1999; Peters et al., 2021). Simple → complex PD maturation might relate to choline transport‐dependent formation of phloem sieve pores (see below) (Dettmer et al., 2014; Kalmbach & Helariutta, 2019; Kraner et al., 2017). It also involves the WD‐repeat protein DECREASED SIZE EXCLUSION LIMIT (DSE1), albeit via unknown mechanisms (Xu et al., 2012). Intriguingly, complex PDs display unusually large cavities in their branching parts in *hpgt123* triple mutant Arabidopsis exhibiting reduced Hyp *O*‐galactosyltransferase (HPGT) activities. This feature aberrantly increases permeability and, hence, symplasmic transport through CWs containing less cellulose and more pectin for unspecified reasons (Okawa et al., 2023).

In addition to aperture, PD number is the second known parameter influencing the extent of symplasmic trafficking and can be controlled dynamically, for example, during flowering transition (Ormenese et al., 2006). PD density‐control raises the fundamental question of how PDs are formed. Primary PDs appear before CW formation via still largely elusive mechanisms (Burch‐Smith et al., 2011; Peters et al., 2021). Recent seminal work indicates that they likely form through ER‐dependent incomplete closure of cell plate fenestrae of dividing cells (Li et al., 2024). Hence, secondary PDs are present between ontogenically related cells. These include the L1‐ or L2‐layer intrinsic cells in apices (Zhang, Chen, & Wang, 2021), or the companion cells (CCs)‐sieve elements (SEs) complex in leaves' phloem (Kim & Frommer, n.d.). At the phloem sieve plates, primary PDs presumably evolve into large sieve pores connecting successive SEs (Kalmbach & Helariutta, 2019; Peters et al., 2021). Sieve pore maturation, possibly within lipid‐raft‐enriched PM nano‐domains (Kalmbach & Helariutta, 2019), requires choline transport by Choline transporter‐like1 (CHER1) (Dettmer et al., 2014) and vesicle trafficking (Gao et al., 2017). Secondary PDs penetrate existing CWs; they may form subsequently to primary PDs within individual layers but exclusively connect ontogenically unrelated cells/layers (Burch‐Smith et al., 2011). Their number increases in tobacco knockdowns of the mitochondrial ISE1 and chloroplastic ISE2 RNA helicases, respectively (Burch‐Smith & Zambryski, 2010; Kobayashi et al., 2007; Stonebloom et al., 2009). The mechanisms connecting secondary PD biogenesis/maturation to ISE1/2, to their known interactors (Bobik et al., 2019) or to their downstream targets in plastidial‐ (Carlotto et al., 2016, 2022) or nuclear‐ gene expression (Ganusova et al., 2020) await full clarification. Nonetheless, plastids' redox status and integrity as well as plastid → nucleus “retrograde” communication are likely important underpinnings of ISE1/2 functions. Conversely, the number of secondary PDs at the epidermis‐mesophyll interface decreases in Arabidopsis *cher1* mutants (Kraner et al., 2017). Yet, whether choline transport or secretory vesicles are involved – as shown during sieve pore maturation – remains unknown.

Unlike primary PDs, secondary PDs form within existing CWs during their extension (Kalmbach & Helariutta, 2019; Seagull, 1983) via processes that are therefore expected to change the CW's biomechanical properties including, chiefly, its stiffness (Brunkard & Zambryski, 2017; Tee & Faulkner, 2024). One key CW component known to influence stiffness is pectin, which is mostly composed of d‐galacturonic acid (GalA) esterified by Golgi‐localized methyl‐ and acetyl‐transferases (Shahin et al., 2023; Shin et al., 2021; Wu et al., 2018). Upon vesicle‐based CW delivery and incorporation, pectin undergoes various degrees and patterns of de‐esterification by CW‐resident pectin acetyl‐ and methyl‐esterases (PAEs; PMEs). The ensuing esterified state influences the degree of CW stiffness according to a model considered applicable to both methyl‐ and acetyl‐esterified homogalacturonan (HG) (Cao et al., 2020; Shin et al., 2021; Wu et al., 2018), a GalA homopolymer accounting for 65% of CW pectin. Its contiguous, block‐wise de‐esterification by plant‐derived (as opposed to fungus‐derived) PAEs/PMEs, for instance, allows HG to form “egg‐box” dimers with Ca^2+^, with the ensuing pectic gel increasing CW stiffness (Cao et al., 2020; Wu et al., 2018). Conversely, loosened CWs contain more esterified HG (Cosgrove, 2016). Strikingly, however, no single factor controlling secondary PD formation has been linked to, or shown to influence, CW‐intrinsic properties to date. The high genetic redundancy of CW‐biosynthetic and ‐modifying enzymes might underpin a spatial or temporal specificity of action perhaps unapproachable by the genetic screens used so far to isolate mutants with broadly altered symplasmic connectivity. These include the aforementioned *ise1‐4* and *dse1* mutants, which, not helping the situation, are all embryo‐lethal.

Silencing small (s)RNAs are among the macromolecules that can move between plant cells. They are composed of (mi)RNAs and small‐interfering (si)RNAs, processed by distinct Dicer‐like (DCL) enzymes from, respectively, imperfect short RNA stem‐loops and perfect long double‐stranded RNAs. Both molecules guide cell‐autonomous ARGONAUTE (AGO) proteins to silence gene expression intracellularly (Bologna & Voinnet, 2014). Non‐loaded, that is, AGO‐free si/miRNA duplexes can also move from cell to cell (Brioudes et al., 2021; Devers et al., 2020) to regulate development among other processes. Endodermis → stele miR165/166 movement, for instance, enables cognate proto (PX)‐ versus metaxylem (MX)‐specification in roots (Carlsbecker et al., 2010). That siRNAs and miRNAs move symplasmically is supported by several lines of indirect or synthetic evidence (Kobayashi & Zambryski, 2007; Liang et al., 2014; Vogler et al., 2008; Voinnet et al., 2000). For example, cells that developmentally lose PDs – such as stomata guard cells (Voinnet et al., 1998) – or cells undergoing synthetic callose deposition at their PDs (Devers et al., 2023; Vatén et al., 2011) neither receive nor emit mobile sRNAs. Transgene‐based silencing reporters and, less frequently, developmental patterning are typically used as readouts in attempts to genetically dissect non‐cell‐autonomous silencing. However, by relying on si/miRNA activity in silencing‐recipient cells, these readouts only provide an indirect indication of mobility. Hence, suppressing or enhancing these readouts is, alone, insufficient to assert effects on si/miRNA cell‐to‐cell movement. Altering cell‐autonomous AGO loading or activity in recipient cells, for instance, would yield similar results (Brioudes et al., 2022). Without additional experiments to tease these possibilities apart reverse genetics has remained indecisive (Fan et al., 2021; Rosas‐Diaz et al., 2018). Forward genetics has failed, likewise, to identify a single sRNA cell‐to‐cell movement factor to date, although an as‐yet‐unexplained role for hydrogen peroxide has been suggested (Liang et al., 2014). Hence, known properties of sRNA mobility remain mostly unexplained, including its contextuality, whereby, under certain circumstances, some tissues are more prone to non‐cell‐autonomous silencing compared with others (reviewed in Voinnet, 2022). sRNA movement may also exhibit directionality at certain cell–cell interfaces (Skopelitis et al., 2018; Voinnet, 2022).

**Figure 1:**
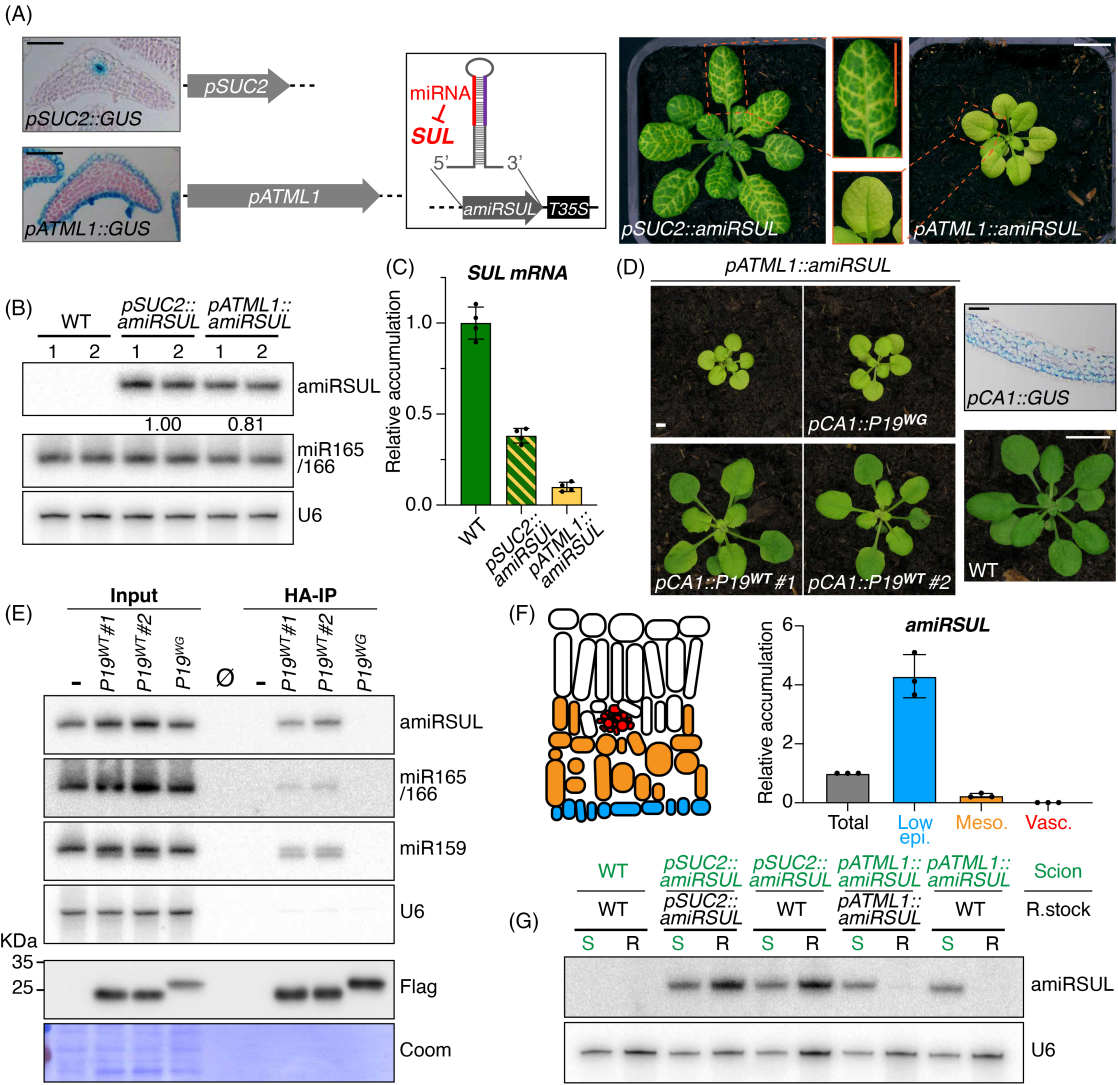
A system to study short‐range cell‐to‐cell miRNA movement via secondary PDs. (A) Schematized constructs and representative rosette/leaf chlorosis for *pSUC2::amiRSUL* and *pATML1::amiRSUL*. Scale bars: 1 cm. Left: companion cell‐ and epidermis‐specific signal from *pSUC2::GUS* and *pATML1::GUS*, respectively, upon leaf cross‐section and GUS staining. Scale bar: 10 μm. T35S: CaMV 35S terminator. (B) amiRSUL northern analysis in WT, *pSUC2::amiRSUL* or *pATML1::amiRSUL* rosette leaves in biological duplicates. miR166/165 and U6 provide RNA loading controls. Relative quantification of the amiRSUL signals, averaged and corrected to endogenous U6, is indicated. Comparable results were obtained for a second independent experiment. (C) RT‐qPCR analysis of *SUL* accumulation in the same tissues as in (B). Error bars: SD. *n* = 4. (D) Phenotype of *pATML1::amiRSUL* rosettes without (−) or with the *pCA1::P19* ^*WT*^ #1, *pCA1::P19* ^*WT*^ #2, or *pCA1::P19* ^*WG*^ transgenes as compared with WT. Scale bar: 1 cm. Upper right: mesophyll‐specific signal from *pCA1::GUS* upon leaf cross‐section and GUS staining. Scale bar: 10 μm. (E) Upper: amiRSUL, miR165/166, and miR159 northern analysis in input and HA‐immunoprecipitated (HA‐IP) fractions from rosette leaves of *pATML1::amiRSUL* co‐expressing the indicated transgenes or not (−). U6: as in (B). Lower: Flag‐based western analysis of P19 alleles. Coomassie blue (Coom) staining provides a protein loading control. ∅: empty lane. Comparable results were obtained for three independent experiments. (F) Left: schematic view of the tissues enriched by Meselect from the abaxial (bottom) side of *pATML1::amiRSUL* rosette leaves. Blue: lower epidermis; orange: mesophyll; red: vasculature. Right: RT‐qPCR analysis of *amiRSUL* accumulation in the Meselect‐enriched fractions indicated on the right. Error bars: SD. *n* = 3. (G) amiRSUL northern analysis in scions (S) or rootstocks (R) in the indicated grafting combinations. U6: as in (B). Comparable results were obtained from two independent experiments.

One explanation for the contemporary lack of genetic insights into silencing cell‐to‐cell movement possibly lies in the very design of the aforementioned sRNA mobility‐readouts. So far, most studies – conducted nearly exclusively in Arabidopsis – have relied upon the mobility of artificial si/miRNAs produced under the CC‐specific promoter *SUCROSE SYMPORTER 2* (*pSUC2*) (Brioudes et al., 2021; Fan et al., 2022; Himber et al., 2003; Smith et al., 2007; Uddin et al., 2014). Targeting photosynthesis‐related mRNAs with such systems, for example, the magnesium chelatase subunit *SUL* mRNA, yields vein‐proximal chlorosis, as seen in the miRNA‐based *pSUC2::amiRSUL* and siRNA‐based *pSUC2::SUL‐LUS* reporters (Brioudes et al., 2021; Himber et al., 2003) (Figure 1A; Figure S1a). *pSUC2*‐based readouts not only rely upon PD connectivity in young leaves but also on CC‐SE translocation of sRNAs from older leaves (Devers et al., 2023), a key and possibly genetically unattainable checkpoint in macromolecular trafficking. Here, we have redesigned a silencing movement reporter to focus solely on communications mediated by secondary PDs in leaves, thereby bypassing the CC‐SE interface. Forward genetics conducted in this new system has allowed identification of a pectin acetyl‐transferase mutation that specifically compromises secondary PD formation, and consequently, si/miRNA, GFP, and virus movement via these channels. These and other results thereby provide the first tangible link between CW biomechanical properties, on the one hand, and PD density, on the other.

## RESULTS

### Probing short‐range cell‐to‐cell silencing movement independently of the CC–SE interface

To bypass the CC–SE contribution to sRNA movement, we developed an alternative mobile silencing‐reporter system by swapping the promoter in *pSUC2::amiRSUL* for the epidermis‐specific promoter *pATML1* (Figure S1a,b; Lu et al., 1996). Transgenic expression of the resulting *pATML1::amiRSUL* caused extensive leaf blade chlorosis as compared with the vein‐restricted chlorosis of the *pSUC2::amiRSUL* transgenic leaves (Figure 1A; Figure S1b). The extensive chlorosis of *pATML1::amiRSUL* correlated in intensity with a reduction in plant stature. Both traits varied proportionally with amiRSUL levels across transformants (Figure S1c,d). Loop‐to‐base versus base‐to‐loop amiRSUL processing from respectively pri‐miR319‐ versus pri‐miR390‐scaffolds yielded the same phenotypic spectrum (Figure S1c–f). Out‐of‐four *pATML1::amiRSUL* T3 lines established from single‐locus transformants, one line with the pri‐miR390‐scaffold accumulated amiRSUL at *pSUC2::amiRSUL*‐comparable levels (Figure 1B) and was selected as a parental line for further experimentation. *SUL* mRNA silencing was stronger in this *pATML1::amiRSUL* line than in *pSUC2::amiRSUL*, as measured by qRT‐PCR (Figure 1C).

Chlorosis caused by *pATML1::amiRSUL* expression was unaltered in siRNA‐deficient *dcl2‐1 dcl3‐1 dcl4‐2* Arabidopsis (Henderson et al., 2006) but abrogated in mutants defective in miRNA biogenesis (*dcl1‐11* and *hyl1‐2*, Han et al., 2004) or activity (*ago1‐42*, Poulsen et al., 2013) (Figure S1g). Thus, chlorosis likely requires amiRSUL production in the lowly photosynthetic epidermis and its non‐cell‐autonomous activity in the subepidermal highly photosynthetic mesophyll. To test this hypothesis, *pATML1::amiRSUL* plants were transformed with either a functional (P19^WT^) or dysfunctional (P19^WG^) version of FLAG‐HA‐epitope‐tagged P19 (Brioudes et al., 2022) expressed under the mesophyll‐specific promoter *pCA1* (Gowik et al., 2004) (Figure 1D,E; Figure S2a). Cell‐autonomous P19 homodimers bind si/miRNA duplexes with high selective affinity, thereby preventing their loading into AGOs (Brioudes et al., 2022). P19‐mediated sequestration of mobile amiRSUL within the mesophyll reduced *SUL* silencing and, hence, chlorosis, as expected (Figure S2b). Two independently established *pATML1::amiRSUL*(*pCA1::P19* ^*WT*^) T3 lines indeed displayed reduced chlorosis, which remained unchanged, by contrast, in *pATML1::amiRSUL*(*pCA1::P19* ^*WG*^) plants (Figure 1D; Figure S2c). HA‐based immunoprecipitation confirmed that amiRSUL was effectively captured by P19^WT^ but not P19^WG^ in mesophyll cells (Figure 1E). Together, these results demonstrate epidermis → mesophyll movement of amiRSUL and suggest that its activity within the mesophyll, not the epidermis, underlies leaf chlorosis. To estimate the extent of amiRSUL movement from the epidermis, we used qRT‐PCR to quantify amiRSUL levels in tissues mechanically separated and enriched via MeSelect (Svozil et al., 2016). Comparing the MeSelect‐isolated lower epidermis versus mesophyll of *pATML1::amiRSUL* rosette leaves revealed a sharp decline in amiRSUL levels (Figure 1F; Figure S2d). Moreover, amiRSUL was below detection in the MeSelect‐isolated vasculature (Figure 1F). These results suggest that amiRSUL moves over a very short distance, thus rationalizing why the veins remain green in *pATML1::amiRSUL* leaves (Figure 1A; Figure S1c,e). Due to its low abundance in the vasculature, we expected amiRSUL long‐distance transport to be restricted. Indeed, amiRSUL was not detected in WT rootstocks grafted onto *pATML1::amiRSUL* scions whereas, as previously reported (Brioudes et al., 2021), it was readily detected in WT rootstocks grafted onto *pSUC2::amiRSUL* scions (Figure 1G). We conclude that *pATML1::amiRSUL* is a suitable reporter of short‐range epidermis → mesophyll miRNA movement in Arabidopsis leaves that bypasses the CC–SE interface involved in *pSUC2*‐based systems.

### *pmr5* partially suppresses silencing without altering amiRSUL biogenesis or intracellular activity

**Figure 2:**
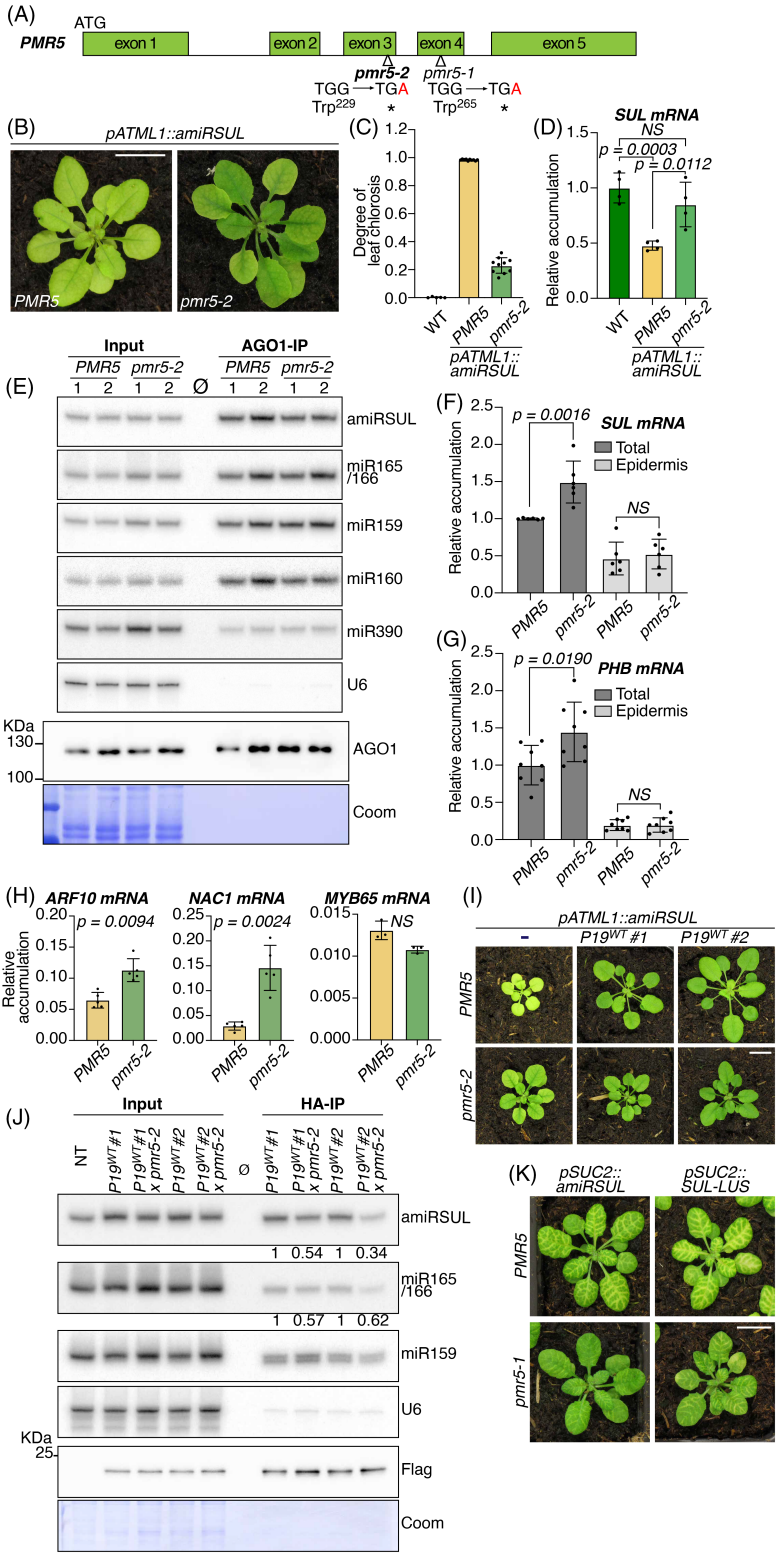
*pmr5* reduces silencing movement without altering amiRSUL biogenesis or intracellular activity. (A) *PMR5* genomic sequence and position of the *pmr5‐1*/*‐2* mutations (arrows). Red font: EMS‐induced nucleotide transitions; *: early stop codon. (B) Phenotype of *PMR5* or *pmr5‐2* rosettes in the *pATML1::amiRSUL* background. Scale bar: 1 cm. (C) Chlorosis quantification in *PMR5* or *pmr5‐2* rosettes in the *pATML1::amiRSUL* background, as compared with WT. Error bars: SD. *n* ≥ 5. (D) RT‐qPCR analysis of *SUL* accumulation in the same tissues as in (C). Error bars: SD. Unpaired two‐tailed *t*‐test *P*‐values are indicated. NS: non‐significant difference. *n* = 4. (E) Upper: amiRSUL, miR165/166, miR159, miR160, and miR390 northern analysis in input and AGO1‐immunoprecipitate (AGO1‐IP) fractions isolated from *PMR5* or *pmr5‐2* rosette leaves in the *pATML1::amiRSUL* background, in biological duplicates. U6 hybridization provides an RNA loading control. Lower: AGO1 western analysis. Coomassie blue (Coom) staining provides a protein loading control. ∅: empty lane. Comparable results were obtained for a second independent experiment. (F) RT‐qPCR analysis of *SUL* accumulation in total or Meselect‐epidermis‐enriched leaf tissues from *PMR5* or *pmr5‐2* in the *pATML1::amiRSUL* background. Error bars: SD. Unpaired two‐tailed test *P*‐value is indicated for total samples. NS as in (D). *n* = 6. (G) Same as (F) but for *PHB* accumulation. Unpaired two‐tailed *t*‐test *P*‐value is indicated. NS is as in (D). *n* = 8. (H) RT‐qPCR analysis of *ARF10* (left, miR160 target), *NAC1* (middle, miR164 target) and *MYB65* (right, miR159 target) accumulation in *PMR5* or *pmr5‐2* rosette leaves in the *pATML1::amiRSUL* background. Error bars: SD. Unpaired one‐tailed *t*‐test *P*‐values are indicated for *ARF10* and *NAC1*. NS as in (D), as obtained by Mann–Whitney two‐tailed test for *MYB65*. *n* ≥ 3. (I) Phenotype of *PMR5* or *pmr5‐2* rosettes in the *pATML1::amiRSUL* background without (−) or with *pCA1::P19* ^*WT*^ transgenes. Scale bar: 1 cm. (J) Upper: amiRSUL, miR165/166, and miR159 northern analysis in input and HA‐immunoprecipitated (HA‐IP) fractions isolated from *pATML1::amiRSUL* leaves co‐expressing the indicated transgenes. U6 RNA hybridization provides RNA loading controls. Relative quantification of the signals, corrected to the endogenous miR159, is indicated. Lower: Flag‐based western analysis of P19 alleles. Coomassie blue (Coom) staining provides a protein loading control. ∅: Empty lane. Comparable results were obtained for two independent experiments. (K) Phenotype of *PMR5* or *pmr5‐1* rosettes expressing the *pSUC2::amiRSUL* or *pSUC2::SUL‐LUS* transgenes. Scale bar: 1 cm.

To identify genes required for miRNA movement, 1500 offspring of EMS‐mutagenized *pATML1::amiRSUL* seeds were germinated on MS plates and seedlings screened for compromised *SUL* silencing. One individual displayed partially suppressed leaf chlorosis (Figure 2A–C) segregating as a single recessive, nuclear trait. Whole‐genome resequencing identified an EMS‐induced G → A transition introducing a premature stop codon within the coding sequence of *POWDERY MILDEW RESISTANCE 5* (*PMR5*) (Figure 2A). *PMR5* encodes a pectin acetyl‐transferase, and accordingly, CWs from the *pmr5* mutant contain less acetyl‐ester (Chiniquy et al., 2019). Reduced F1 chlorotic phenotypes revealed allelism between the G → A transition and the *pmr5‐1* null allele previously isolated in a screen for enhanced resistance to powdery mildew albeit via as‐yet‐unexplained mechanisms (Figure 2A; Figure S3a; Vogel et al., 2004). Therefore, we named the identified mutant *pmr5‐2* (Figure 2A,B; Figure S3a). Upon introgression into the *pATML1::amiRSUL* parental line [generating *pATML1::amiRSUL* ^*(pmr5‐1)*^], *pmr5‐1* partially suppressed chlorosis, phenocopying *pmr5‐2* (Figure S3b). Based on these results and on the comparable effects of both alleles on rosettes' phenotypes (reduction of the rosette size, flatter leaves; Figure S3c,d), *pmr5‐1* and *pmr5‐2* were thus used interchangeably in further experiments conducted in this study.

The *pATML1::amiRSUL* parental line has a smaller stature compared with WT correlating with extensive leaf chlorosis, suggesting that due to *SUL* silencing, plants do not produce sufficient chlorophyll, resulting in impaired growth (Figure 1C; Figures S1g; Figure S2c). Despite non‐transgenic *pmr5‐1* and *pmr5‐2* also displaying some growth defects (Figure S3c; Vogel et al., 2004), the rosette size of *pATML1::amiRSUL* ^*(pmr5‐1)*^ and *pATML1::amiRSUL* ^*(pmr5‐2)*^ plants was ameliorated in comparison to *pATML1::amiRSUL* (Figure S3b). In addition, chlorosis was reduced and stature increased when *pmr5‐1* was introgressed into a low‐expressing *pATML1::amiRSUL* line [Figure S3e,f; *line pATML1::amiRSUL*(*low*)], making it unlikely, therefore, that *pmr5‐1* growth defects *per se* impede mobile silencing (Figure S3e,f). Confocal microscopy of the *pPMR5::PMR5‐GFP* construct, previously shown to correct *pmr5‐1* morphological defects and to restore its fungal susceptibility (Chiniquy et al., 2019), confirmed PMR5's endoplasmic reticulum association in the epidermis and presence in the underlying mesophyll and vascular tissues (Figure S3g,h). Furthermore, when re‐engineered, transgenic expression of the *pPMR5::PMR5:GFP* construct in the *pmr5‐2* background restored leaf chlorosis (Figure S3g). We conclude from these allelism and complementation assays that *pmr5* is causative of the suppressed amiRSUL‐mediated chlorosis independently of growth defects' intensity.

Consistent with their partial reversal of silencing, *pATML1::amiRSUL* ^*(pmr5‐2)*^ leaves accumulated higher *SUL* mRNA levels than in *pATML1::amiRSUL* (Figure 2D). Reduced silencing was not caused by impaired miRNA production because amiRSUL and endogenous (endo)miRNA levels were unchanged in *pATML1::amiRSUL* ^*(pmr5‐1)*^ *or pATML1::amiRSUL* ^*(pmr5‐2)*^ compared with *pATML1::amiRSUL* (Figure 2E, Input lanes; Figure S4a). This result was extended to all annotated Arabidopsis miRNAs in sRNA‐Seq analyses comparing each *pmr5* allele to WT (Figure S4b). Furthermore, in both *pATML1::amiRSUL* and *pATML1::amiRSUL* ^*(pmr5‐2)*^, amiRSUL and endo‐miRNAs were similarly loaded into AGO1, the main Arabidopsis effector of miRNAs including amiRSUL (Figure 2E; Figure S1g). Likewise, *SUL* mRNA accumulation was similar in the MeSelect‐isolated epidermis of both genotypes, suggesting intact AGO1‐dependent amiRSUL activity within amiRSUL‐producing cells (Figure 2F; Figure S4c). To further verify that AGO1 intracellular activity is unimpeded, we tested constitutive AGO1‐dependent RNAi of *CHALCONE SYNTHASE* (*CHS*) (Jay et al., 2011). Low *CHS* mRNA accumulation in leaves and lack of seed coat pigmentation, a phenotype caused by *CHS* silencing in WT, remained unchanged in *pmr5‐1* (Figure S4d). We conclude that defective amiRSUL‐biogenesis, ‐stability, ‐loading, or impaired AGO1 intracellular activity cannot explain the reduced chlorosis of *pATML1::amiRSUL* ^*(pmr5‐2)*^.

### PMR5 enables sRNA movement and symplasmic macromolecular trafficking tissue‐dependently

Unlike in the mechanically isolated amiRSUL‐producing epidermis, *SUL* mRNA accumulation was increased in whole leaves of *pATML1::amiRSUL* ^*(pmr5‐2)*^ compared with *pATML1::amiRSUL* (Figure 2F; Figure S4c). That silencing defects were thus apparently confined to amiRSUL‐recipient cells suggested roles for PMR5 in intercellular movement of amiRSUL, and possibly of endo‐miRNAs. Indeed, a similar epidermis‐versus‐whole leaf disparity affected the levels of *PHABULOSA* (*PHB*), targeted by mobile miR165/166 produced from the lower epidermis (Zhu et al., 2011) (Figure 2G; Figure S4c). *AUXIN RESPONSE FACTOR10* (*ARF10*) and *NAC DOMAIN‐CONTAINING PROTEIN1* (*NAC1*) –known targets of mobile miR160 and miR164 (Brosnan et al., 2019) – also had increased levels in *pmr*5*‐2*‐ versus WT‐whole leaves (Figure 2H). This was unlike multiple targets of endo‐miRNAs not reported as mobile, including *MYB DOMAIN PROTEIN65* (*MYB65*) regulated by the near‐ubiquitous miR159 (Li et al., 2016; Figure 2H; Figure S4e). *pmr5‐2* was introgressed into the two independent *pATML1::amiRSUL*(*pCA1::P19* ^*WT*^) lines used to validate amiRSUL epidermis → mesophyll movement (Figure 1D,E). Chlorosis was further reduced in the resulting *pATML1::amiRSUL*(*pCA1::P19* ^*WT*^)^*(pmr5‐2)*^ plants in which amiRSUL was consistently less co‐immunoprecipitated (co‐IPed) with mesophyll‐expressed P19 than in *pATML1::amiRSUL*(*pCA1::P19* ^*WT*^) plants (Figure 2I,J). Mobile miR165/166 also was less co‐IPed (Figure 2J). These key experiments and results therefore establish unambiguously that PMR5 enables cell‐to‐cell movement of amiRSUL and at least some endo‐miRNAs from the mesophyll to the epidermis. Consistent with PMR5:GFP vascular accumulation in leaves (Figure S3g), *pmr5‐1* also reduced silencing from *pSUC2::amiRSUL* and from the related siRNA‐based *pSUC2::SUL‐LUS* reporter (Himber et al., 2003) although vein‐centered chlorosis was still clearly visible in both (Figure 2K; Figure S4f,g). Thus, *pmr5* effects on silencing in leaves are neither epidermis‐ nor miRNA‐specific.

**Figure 3:**
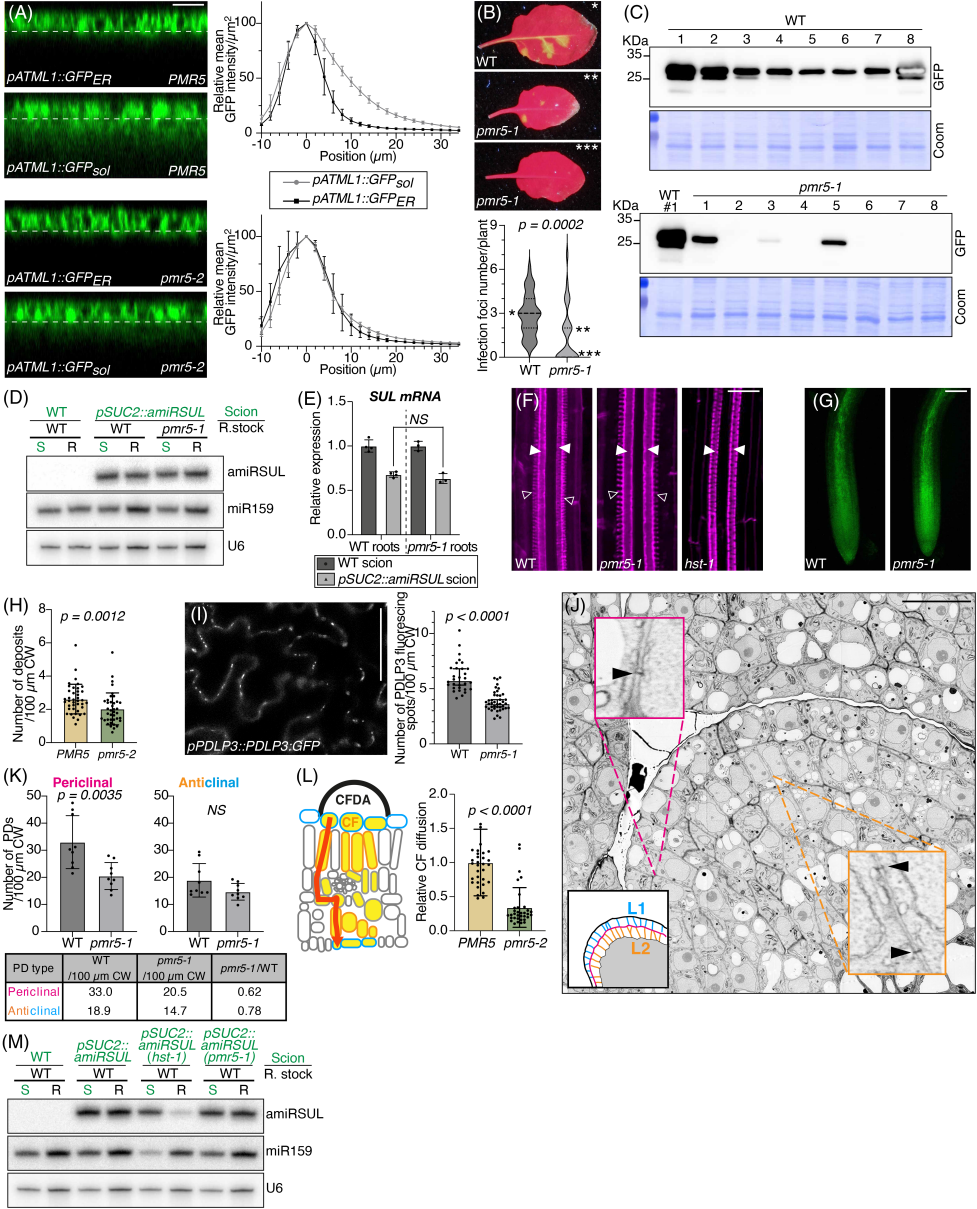
PMR5 enables symplasmic trafficking by facilitating secondary PD formation in a tissue‐dependent manner. (A) Left: orthogonal views of confocal stack images from the adaxial epidermis in young seedlings' leaves of *pATML1::GFP*_*ER*_ or *pATML1::GFP*_*sol*_ in *pmr5‐2* or *PMR5*, in the *pATML1::amiRSUL* background. ER: endoplasmic reticulum‐targeted; sol: soluble, free‐GFP. Scale bar: 20 μm. Full images are provided in Figure S5(b). Right: relative and centered mean GFP intensities in relation to the position's depth in the indicated genotypes, in the *PMR5* (up) or *pmr5‐2* (bottom) backgrounds. Error bars: SD. *n* = 10. (B) Imaging (up) and quantification (bottom) of primary infection foci 5 days post‐TuMV‐GFP inoculation in the indicated genotypes. Error bars: SD. *n* = 30. Data are corrected by ratio (size pmr5 = 0.57 × WT, Vogel et al., 2004). Images are not representative of the quantification but illustrate when no infection foci are observed in *pmr5‐1* (***) or how restricted the TuMV‐GFP foci and vasculature infection appear in *pmr5‐1* (**) as compared with WT (*). (C) GFP western analysis reporting TuMV‐GFP accumulation in rosette leaves 7 days post‐TuMV‐GFP inoculation of WT or *pmr5‐1* plants. Coomassie blue (Coom) staining provides a protein loading control. Comparable results were obtained for a second independent experiment. (D) amiRSUL northern analysis in scions (S) and rootstocks (R) in the indicated grafting combinations. miR159 and U6 provide RNA loading controls. Comparable results were obtained for a second independent experiment. (E) RT‐qPCR analysis of *SUL* accumulation in roots of the indicated grafting combinations. Error bars: SD. Unpaired two‐tailed *t*‐test result is indicated. NS: non‐significant difference. *n* = 3. (F) Basic fuchsin staining of protoxylem (unfilled arrowheads) and metaxylem (filled arrowheads) in WT, *pmr5‐1*, and *hst‐1* roots. Scale bar: 20 μm. (G) Confocal imaging of free‐GFP signals in roots of *pSUC2::GFP*_*sol*_ in the indicated genotypes. Scale bar: 100 μm. (H) Quantification of aniline blue deposits per 100 μm CW in *PMR5* or *pmr5‐2* rosette leaves in the *pATML1::amiRSUL* background. Error bars: SD. Mann–Whitney two‐tailed test *P*‐value is indicated. *n* ≥ 39. Comparable results were obtained for two independent experiments. (I) Left: confocal imaging of *pPDLP3::PDLP3:GFP* signal in WT leaf epidermal cells. Scale bar: 50 μm. Right: quantification of the number of PDLP3‐GFP spots per 100 μm CW in rosette leaves in the WT or *pmr5‐1* background. Error bars: SD. Mann–Whitney two‐tailed test *P*‐value is indicated. *n* ≥ 30. (J) Scanning electron microscopy inverted image of a *pmr5‐1* shoot apical meristem (SAM) illustrating the positions of periclinal L1‐to‐L2 secondary PDs (magnification delimited in pink) or anticlinal L1‐ and L2‐monolayer‐intrinsic primary and secondary PDs (magnification delimited in orange). Scale bar: 10 μm. Left bottom: schematic localization of L1 and L2. (K) Quantification of the number of periclinal (left) and anticlinal (right) PDs in WT or *pmr5‐1* SAMs. Error bars: SD. Unpaired two‐tailed *t*‐test *P*‐value is indicated for periclinal quantifications. Mann–Whitney two‐tailed test gives non‐significant difference (NS) for anticlinal quantifications. *n* = 9. (L) Left: leaf adaxial‐to‐abaxial symplasmic CF diffusion involves crossing of several distinct cell types (yellow) via PDs (red). Right: quantified relative CF diffusion in rosette leaves of the indicated genotypes. Error bars: SD. Mann–Whitney two‐tailed test *P*‐value is indicated. *n* ≥ 32. Comparable results were obtained for two independent experiments. (M) amiRSUL and miR159 northern analysis in scions (S) and rootstocks (R) in the indicated grafting combinations. U6: same as (D). Comparable results were obtained for two independent experiments.

To assay other macromolecules' cell‐to‐cell movement, we introgressed *pmr5‐1* into two previously established lines. In *pSUC2::*_*tm*_ *GFP9*, a membrane‐anchored allele of GFP (_tm_GFP9) is CC‐restricted in mature leaves (Stadler et al., 2005) (Figure S5a). In *pSUC2::GFP*_*sol*_, free‐GFP undergoes CC → SE translocation, is phloem‐transported and is unloaded in young leaves' laminae where it diffuses symplasmically (Imlau et al., 1999) (Figure S5a). While the _tm_GFP9 pattern in *pSUC2::*_*tm*_ *GFP9* remained unaltered, free‐GFP diffusion was consistently less extensive in young leaves' blade of *pSUC2::GFP*_*sol*_^*(pmr5‐1)*^‐compared with *pSUC2::GFP*_*sol*_‐seedlings (Figure S5a). To more quantitatively assess impairment of free‐GFP movement, we engineered line *pATML1::GFP*_*sol*_ in which epidermal free‐GFP diffused symplasmically into young seedlings' mesophyll over up to ~20 μm (Figure 3A; Figure S5b). *pATML1::GFP*_*ER*_, in which ER‐targeted (_ER_GFP) accumulation is epidermis‐restricted, was also constructed. Free‐GFP diffusion, but not the _ER_GFP pattern, was strongly compromised in *pATML1::GFP*_*sol*_^*(pmr5‐2)*^ leaves (Figure 3A; Figure S5b,c). We also tested active transport mediated by a viral movement protein (MP). Upon inoculation of WT Arabidopsis leaves, GFP‐tagged turnip mosaic virus (TuMV‐GFP) produces expanding foci that eventually reach the vasculature, reflecting MP‐mediated trafficking across diverse cell types. Upon inoculation of *pmr5‐1* or *pmr5‐2* mutant leaves, the primary infection foci were either absent or smaller, and less numerous than in WT‐inoculated leaves (Figure 3B; Figure S6a). Moreover, if formed, they generally failed to reach the veins, resulting in low infection frequencies assessed in whole plants (Figure 3B,C; Figure S6b). We conclude that PMR5 facilitates both passive (free‐GFP) and active (TuMV‐GFP) cell‐to‐cell movement in leaves.

We tested if PMR5 is required in roots where PMR5:GFP accumulates at shoot‐like levels in complemented *pPMR5::PMR5:GFP* ^*(pmr5‐1)*^ plants (Figure S6c). amiRSUL was efficiently graft‐transmitted from *pSUC2::amiRSUL* scions into both *pmr5‐1* and WT rootstocks (Figure 3D); both displayed similar *SUL* silencing efficacies (Figure 3E), suggesting comparable amiRSUL phloem unloading and cell‐to‐cell movement. Likewise, PX versus MX specification, enabled by endodermis → stele miR165/166 movement, occurred normally in the *pmr5‐1* stele, unlike, as reported, in the stele of *hst‐1*, which carries a mutation in *HASTY* required for cellular emission of mobile miRNAs including amiRSUL (Figure 3F; Brioudes et al., 2021). Moreover, free‐GFP phloem unloading and cell‐to‐cell symplasmic movement were as extensive in *pSUC2::GFP*_*sol*_^*(pmr5‐1)*^ – as they were in *pSUC2::GFP*_*sol*_ – root tips (Figure 3G; Figure S6d). Therefore, *pmr5‐1* effects on symplasmic movement are tissue dependent.

### PMR5 controls PD number between epidermal and subepidermal cell layers and facilitates symplasmic connectivity in leaves

Because plant si/miRNAs, proteins, and viruses move symplasmically, *pmr5* might reduce PD aperture and/or number. Given the TuMV‐GFP results (Figure 3B,C; Figure S6a), the former possibility was less likely because viral MPs can enlarge low‐aperture PDs (Kumar & Dasgupta, 2021). The number of CW‐embedded aniline blue deposits, marking callose‐associated PDs, was reduced by 23% in *pATML1::amiRSUL* ^*(pmr5‐2)*^‐compared with *pATML1::amiRSUL‐*epidermal monolayers comprising both primary and secondary PDs (Figure 3H). Similar results were obtained in *pmr5‐1* (Figure S6e). Assessing PD number callose‐independently in *pPDLP3::PDLP3:GFP* ^*(pmr5‐1)*^‐ versus *pPDLP3::PDLP3:GFP*‐epidermal monolayers yielded similar results, supporting a role for PMR5 in controlling PD frequency (Figure 3I). In scanning electron microscopy, the number of periclinal PDs connecting the L1‐to‐L2 monolayers – composed exclusively of secondary PDs – was reduced by 38% in *pmr5‐1*‐ versus WT‐shoot apical meristems (Figure 3J,K). This agreed with the partial *pmr5‐2* effects in *pATML1::amiRSUL* (Figure 2B), where very short‐range L1 → L2 amiRSUL movement via mostly secondary PDs likely underlies chlorosis (Figure 1F). The number of anticlinal PDs, that is, between cells within the L1‐ or L2‐monolayers, comprising both primary and secondary PDs, was also reduced in *pmr5‐1* (Figure 3J,K), albeit just below statistical significance suggesting, altogether, that *pmr5* predominantly impacts secondary PDs.

Carboxyfluorescein diacetate (CFDA) is a non‐fluorescent membrane permeable dye that is cleaved once inside plant cells to fluorescent carboxyfluorescein (CF), as illustrated in stomata cells upon adsorption of CFDA (Cui et al., 2015; Lee et al., 2011; Figure S6f, left panel). CF is membrane impermeable and diffuses through plant tissues in a strict symplastic manner; thus, it is excluded from abaxial leaf stomata after its adaxial → abaxial diffusion (Figure S6f, right panel). Therefore, we measured the symplastic diffusion of CF in WT or *pmr5* leaves to ascertain *pmr5* effects on secondary PDs and simultaneously assess *pmr5* impact on long‐range cell‐to‐cell movement. Because CF must traverse many distinct cell types mostly connected via secondary PDs (Figure 3L), strong *pmr5* effects were expected. Indeed, CF diffusion was reduced by 67% in *pATML1::amiRSUL* ^*(pmr5‐2)*^ versus *pATML1::amiRSUL* leaves (Figure 3L). In *pSUC2*‐based reporters, chlorosis depends on cell‐to‐cell diffusion of sRNAs via both primary and secondary PDs upon their phloem unloading in young leaves (Devers et al., 2023). However, this only occurs upon prior translocation of sRNAs from CCs to SEs in mature leaves (Devers et al., 2023), with the latter underpinning phloem‐based long‐distance transport, including during grafting. Being ontogenically related, CCs and SEs are mostly connected via primary, not secondary PDs. That PMR5 affects secondary PDs more specifically therefore predicted that amiRSUL graft transmission into WT rootstocks would be equally efficient from either *pSUC2::amiRSUL* ^*(pmr5‐2)*^‐ or *pSUC2::amiRSUL*‐scions. This was indeed the case, whereas amiRSUL graft transmission was abrogated from *pSUC2::amiRSUL* ^*(hst‐1)*^ scions, as reported (Brioudes et al., 2021) (Figure 3M). Together, these results identify a hitherto unknown role for PMR5 in controlling secondary PD frequency between epidermal and subepidermal cell layers, without overtly impacting CC–SE connectivity.

### Pectin modifications impact symplasmic connectivity in leaves

**Figure 4:**
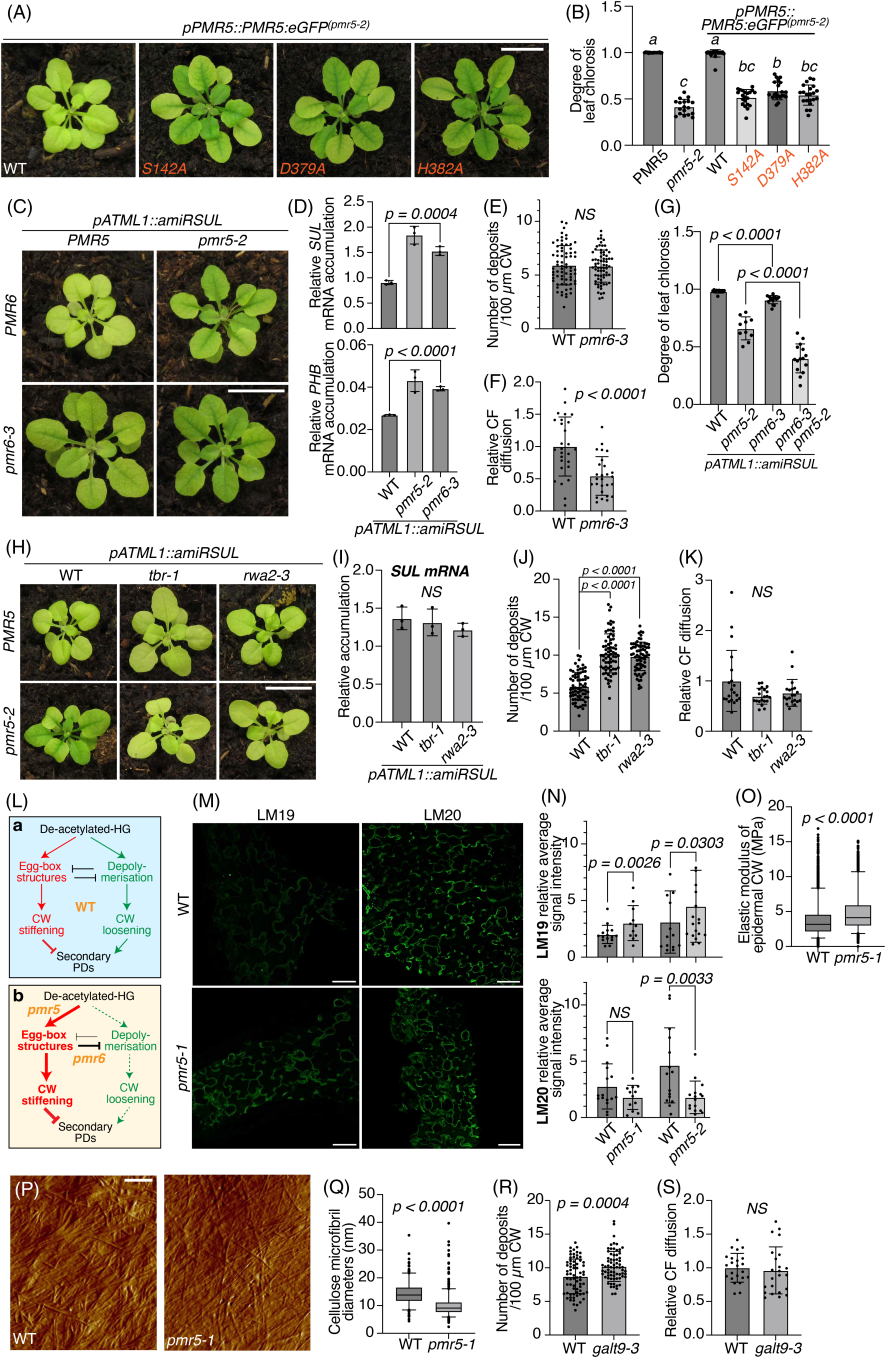
Pectin de‐esterification and leaf CW stiffness correlate with altered PD formation. (A) Phenotype of *pmr5‐2* rosettes in the *pATML1::amiRSUL* background complemented with a WT or point mutated versions (S142A, D379A or H382A) of *pPMR5::PMR5:eGFP*. Scale bar: 1 cm. (B) Leaf chlorosis quantification in the indicated genotypes in the *pATML1::amiRSUL* background. *n* ≥ 18. a, b, c: compact letter display of the Dunn's test of multiple comparisons. (C) Phenotype of simple or double *pmr5‐2 pmr6‐3* rosettes in the *pATML1::amiRSUL* background. Scale bar: 1 cm. (D) qRT‐PCR analysis of *SUL* (up) and *PHB* (bottom) accumulations in rosette leaves of *pmr5‐2* or *pmr6‐3* as compared with WT, in the *pATML1::amiRSUL* background. Error bars: SD. Unpaired two‐tailed *t*‐test *P*‐values are indicated. *n* = 3. (E) Quantification of aniline blue deposits per 100 μm CW in rosette leaves of *pmr6‐3* compared with WT. Error bars: SD. Unpaired two‐tailed *t*‐test *P*‐value is indicated. *n* = 70. (F) Relative CF diffusion in the same tissues as in (E). Error bars: SD. Unpaired two‐tailed *t*‐test *P*‐value is indicated. *n* = 27. Comparable results were obtained from one independent experiment. (G) Leaf chlorosis quantification in the indicated genotypes, in the *pATML1::amiRSUL* background. Error bars: SD. Mann–Whitney two‐tailed test (*pmr6‐3* versus WT) or unpaired two‐tailed *t*‐test (*pmr5‐2 pmr6‐3* versus *pmr5‐2*) *P*‐values are indicated. *n* ≥ 8. (H) Phenotype of simple *tbr‐1*, *rwa2*‐*3* or double *tbr‐1 pmr5‐2*, *rwa2‐3 pmr5‐2* versus WT rosettes, in the *pATML1::amiRSUL* background. Scale bar: 1 cm. (I) RT‐qPCR analysis of *SUL* accumulation in rosette leaves of *tbr‐1* or *rwa2‐3* as compared with WT, in the *pATML1::amiRSUL* background. Error bars: SD. Unpaired two‐tailed *t*‐tests give non‐significant differences (NS). *n* = 3. (J) Quantification of aniline blue deposits per 100 μm CW in rosette leaves of *tbr‐1* or *rwa2‐3* as compared with WT. Error bars: SD. Unpaired two‐tailed *t*‐test *P*‐values are indicated. *n* = 70. (K) Relative CF diffusion in the same tissues as in (J). Error bars: SD. Mann–Whitney two‐tailed tests give non‐significant differences (NS). *n* = 20. Comparable results were obtained for one independent experiment. (L) Temptative model of the de‐acetylated‐homogalacturonan (HG)'s fate in the WT (up), *pmr5* or *pmr6* (bottom) cell walls (CWs). Increased CW stiffness might result from a decreased acetylated state (*pmr5*) or reduced HG depolymerisation (*pmr6*). (M) Confocal images of WT or *pmr5‐1* cotyledon epidermal monolayers showing LM19 (left) or LM20 (right) signals. Scale bar: 50 μm. (N) Quantifications of the relative average of LM19 (upper panel) and LM20 (bottom panel) signal intensities obtained on *pmr5‐1*, *pmr5‐2* cotyledon epidermal monolayers as compared with their respective WT control. Mann–Whitney one‐tailed *P*‐values are indicated. NS: non‐significant difference. *n* ≥ 11. (O) Quantification of the elastic modulus of WT or *pmr5‐1* cotyledon epidermal CW measured by atomic force microscopy. Mann–Whitney one‐tailed test *P*‐value is indicated. *n* ≥ 3643. (P) Representative images of cellulose microfibrils in the same tissues as (O) observed by atomic force microscopy. Scale bars: 250 nm. (Q) Cellulose microfibrils' diameter quantification in the same tissues as (O). Mann–Whitney two‐tailed test *P*‐value is indicated. *n* ≥ 340. (R) Quantification of aniline blue deposits per 100 μm CW in rosette leaves of *galt9‐3* as compared with WT. Error bars: SD. Unpaired two‐tailed *t*‐test *p*‐value is indicated. *n* = 70. Comparable results were obtained for one independent experiment. (S) Relative CF dye diffusion in the same tissues as in (R). Error bars: SD. Mann–Whitney two‐tailed test gives non‐significant difference (NS). *n* = 23. Comparable results were obtained for one independent experiment.

How might PMR5 influence PD frequency in leaves? PMR5 displays amino acid sequence and *in vitro* activities of a pectin acetyl‐transferase via a highly conserved PMR5/DUF231 catalytic triad (Chiniquy et al., 2019). None of the three point mutations in this catalytic domain rescued fungal susceptibility in *pmr5‐1* (Chiniquy et al., 2019). Similarly, none rescued leaf chlorosis in *pATML1::amiRSUL* ^*(pmr5‐2)*^ when introduced into a genomic GFP fusion expressed under the *pPMR5* promoter (Figure 4A,B) to the same extent as the WT PMR5 version. Since the three mutant alleles accumulated comparably to the WT complementing allele (Figure 4A,B; Figure S7a), we conclude that mobile amiRSUL‐mediated silencing requires PMR5 enzymatic activity presumably via pectin acetylation. Strikingly, the screen that isolated *pmr5‐1* also identified mutant alleles of *POWDERY MILDEW RESISTANCE 6* (*PMR6*) which, consistent with increased pectin levels in *pmr6* CWs, encodes a putative pectate lyase‐like (PLL; Vogel et al., 2002), a class of enzymes known to mediate pectin depolymerisation (Shahin et al., 2023; Shin et al., 2021; Wu et al., 2018). When introgressed into *pATML1::amiRSUL*, *pmr6‐3* partially suppressed chlorosis, *SUL*‐ and *PHB*‐silencing, albeit less than *pmr5‐2* (Figure 4C,D); *pSUC2::amiRSUL* ^*(pmr6‐3)*^ and *pSUC2::SUL‐LUS* ^*(pmr6‐3)*^ plants also displayed moderately reduced vein‐centered chlorosis (Figure S7b–e). Like in *pmr5 leaves*, *PHB*, *ARF10*, and *ARF17* accumulated more in *pmr6‐3* leaves, unlike *MYB65* (Figure S7f). PD‐associated callose deposits in leaf epidermal monolayers were statistically unchanged (Figure 4E), whereas adaxial → abaxial CF diffusion was reduced by 46% in *pmr6‐3* compared with 66% in *pmr5‐2* (Figure 4F compared with Figure 3L). Finally, amiRSUL‐dependent chlorosis was more reduced in the *pmr5‐2 pmr6‐3* double‐mutant than in either single‐mutant leaves (Figure 4C,G), evoking how the mutations' synergistic effects on altered CW composition previously advocated their involvements in parallel pectin‐related pathways (Vogel et al., 2004). Together, these results suggest that pectin acetylation by PMR5 or depolymerization of de‐esterified pectin by PMR6 can independently and additively facilitate symplastic movement in leaves.

A secondary screen for restored fungal susceptibility in *pmr5‐1* identified suppressor mutations in *REDUCED WALL ACETYLATION‐2* (*RWA2*) and *TRICHOME BIREFRINGENCE* (*TBR*) (Chiniquy et al., 2019). RWA2 mediates xyloglucan and xylan acetylation perhaps competitively to pectin under possibly limiting O‐acetyl donors' levels (Shahin et al., 2023). TBR, a DUF231 protein like PMR5, is thought to limit pectin de‐acetylation by PAEs (Sinclair et al., 2017) and, possibly, moderate hypo‐acetylation in *pmr5*. Further underscoring the emerging link between pectin, CW and PDs, both *tbr‐1* and *rwa2‐3* restored leaf chlorosis upon their introgression into *pATML1::amiRSUL* ^*(pmr5‐2)*^ plants (Figure 4H; Figure S7g). Neither phenotype was overtly enhanced in *pATML1::amiRSUL* ^*(tbr‐1)*^ or *pATML1::amiRSUL* ^*(rwa2‐3)*^ plants, however (Figure 4H,I; Figure S7g), despite their epidermal monolayer accumulating more PD‐associated callose deposits than in WT plants (Figure 4J). This suggested that increasing, as opposed to decreasing, secondary PD frequency in leaves does not overtly impact mobile amiRSUL silencing or symplasmic movement in general, because abaxial → adaxial CF diffusion was not enhanced in *tbr‐1* or *rwa2‐3* compared with WT (Figure 4K).

### PMR5 provides a potential link between CW loosening, CW extension, and secondary PD frequency

Both *pmr5* and *pmr6* have reduced leaf and cell size ascribed to defective cell expansion (Vogel et al., 2002, 2004). CW extension, during which secondary PDs form (Kalmbach & Helariutta, 2019; Seagull, 1983), underpins cell expansion and requires CW loosening (Wu et al., 2018). We reasoned that a fraction of non‐acetylated pectin incorporating the *pmr5* CW might become more available to Ca^2+^‐dependent gelation, which increases CW stiffness. This would possibly impede CW extension, and hence, reduce secondary PD number (Figure 4L). To assess pectin esterification in *pmr5*, the HG‐specific LM19 and LM20 monoclonal antibodies (Verhertbruggen et al., 2009) were used to probe periclinal CW monolayers isolated from cotyledons' epidermis, the leaf layer in which PMR5‐dependent L1 → L2 movement of amiRSUL and free‐GFP was established. Unlike LM20, LM19 preferentially and strongly binds to low‐ or non‐methylated HG (Verhertbruggen et al., 2009). Immunofluorescence‐based quantifications under the confocal microscope showed that LM19 yields a substantially higher signal in both *pmr5‐1* and *pmr5‐2* than in WT CWs (Figure 4M,N; Figure S7h). By contrast, the LM20 signal intensity was reduced in the two *pmr5* alleles, as compared with their WT controls, even though the difference was only statistically significant for the *pmr5‐2* allele (Figure 4M,N; Figure S7h). These results suggest that HG is less methylated in *pmr5* and might thus have a greater propensity to form egg box dimers with Ca^2^, as inferred, with the same antibody set, in multiple plant species (Frey et al., 2023; Parra‐Rojas et al., 2023).

To directly assess CW stiffness, we applied atomic force microscopy to periclinal CW monolayers also isolated from cotyledons' epidermis. The elastic modulus was higher in *pmr5‐1* than in WT (Figure 4O), revealing a stiffer epidermal CW in the mutant. Moreover, and perhaps counter‐intuitively, the diameter of cellulose microfibrils was nearly halved in *pmr5‐1* compared with WT CWs, unraveling structural, in addition to mechanical defects deserving further investigation (Figure 4P,Q). To further ascertain a potential link between CW stiffness and PD number, we used a mutation in the galactosyltransferase‐encoding gene *GALT9*, which controls GalA abundance. Due to reduced CW pectin availability, the CW extension and cell size increase in *galt9* correlate with a lower CW elastic modulus compared with WT (Zhang, Guo, et al., 2021), the converse effect of *pmr5* (Figure 4L). Accordingly, the PD number, estimated by the density of callose deposits, was increased by 18% in *galt9‐3* compared with WT epidermal monolayers (Figure 4R; Figure S7i). However, adaxial → abaxial CF diffusion was not enhanced in *galt9‐3* compared with WT (Figure 4S), consistent with the results obtained with the *tbr‐1* and *rwa2‐3* suppressors of *pmr5* (Figure 4J,K). Collectively, these results support the notion that PMR5 enables CW loosening as a prerequisite to CW extension required for secondary PD formation.

## DISCUSSION

PMR5 is the first factor isolated to date that affects physical trafficking of si/miRNAs between adjacent cells, and one among very few known to influence the poorly understood processes of PD formation. While reduced in stature, *pmr5* mutants remain viable and fertile, contrasting with the embryo‐ or seedling‐lethality of nearly all Arabidopsis PD mutants isolated so far. Thus, the *pmr5* background might provide a useful probe of suspected PD‐mediated processes, at least in aerial tissues. For instance, use of *pmr5* may help ascertain the trafficking route of the homeodomain transcription factor KNOTTED1 (KN1), of which the mesophyll → epidermis movement was reconstructed in the Arabidopsis shoot meristem (Kitagawa et al., 2022). Noteworthy, both the KN1 protein and *KN1* transcripts move in the assay, which could help assess the requirement for PMR5 in mRNA cell‐to‐cell movement, an aspect not tested here. A decreased PD number explains why symplasmic cell‐to‐cell movement was generally impeded in *pmr5*, affecting both passive and active trafficking of the dye and macromolecules tested. This unanticipated link with symplasmic movement might provide a fresh perspective to elucidate the so‐far mysterious underpinnings of *pmr5's* enhanced resistance to powdery mildew (Vogel et al., 2004). This idea is supported by the systematically converging effects of *pmr6*, *pmr5* and its suppressors on fungal susceptibility, on the one hand, and chlorosis mediated by mobile amiRSUL, on the other. *pmr5's* effects on PD frequency or amiRSUL movement were respectively less (Figure 3H–K) or not (Figure 3M) visible under settings involving primary PDs which, unlike secondary PDs, develop before CW formation. A priori not affected by the CW‐modifying *pmr5*, *pmr6* or *galt9* mutations, primary PDs are structurally undistinguishable from secondary PDs. Thus, the seemingly modest effects of the above mutations within L1 or L2 monolayers – in which both PD types were scored – are likely explained by dilution. Collectively, our results suggest that pectin‐mediated modulation of CW stiffness is one possible mechanism influencing secondary PD frequency in a manner linked to CW extensibility. Measuring CW stiffness in PAE‐overexpressing Arabidopsis might further illuminate the influence of pectin acetylation. However, our results suggest that this control is achievable not only by endomembrane‐based pectin acetylation (PMR5), but also by mere pectin availability (GALT9) or CW‐based depolymerization of de‐esterified pectin (PMR6). By promoting HG depolymerization, PMR6‐like PLLs might compete with PMR5's action. Under certain circumstances, protons released during pectin depolymerization can stimulate CW‐degrading enzymes causing loosening (Wu et al., 2018). We thus speculate that unlike in *pmr5*, fully acetylated pectin might incorporate the *pmr6* CW where it would, however, undergo less depolymerization (Figure 4L). This would possibly leave more pectin available for gelation‐induced CW stiffness, incidentally rationalizing the *pmr5‐1 pmr6‐3* additive effects (Figure 4L). We do not discount, however, the additional possibility that pectins with specific properties, influenced by PMR5, might be required for secondary PD formation/stabilization at particular CW microdomains (Dauphin et al., 2022). Consistent with a role for pectin in PD formation/biology, specific pectin signatures were found concentrated on secondary PD aggregates called pit fields (Faulkner et al., 2008; Orfila & Knox, 2000). Additionally, pectin‐modifying enzymes are found in the Arabidopsis plasmodesmal proteome (Fernandez‐Calvino et al., 2011).

Noteworthy, reduced vein‐centered chlorosis in *pSUC2::amiRSUL* and *pSUC2::SUL‐LUS* with the *pmr5* (Figure 2K) or *pmr6* (Figure S7b,d) background was still readily visible in ways that would have likely evaded forward screening. Indeed, unlike in *pATML1::amiRSUL*, key aspects of silencing movement in these reporters rely upon primary PDs in addition to, or independently of, secondary PDs (Devers et al., 2023). While, in hindsight, the reporters' influence was perhaps predictable, our study additionally revealed a less foreseeable impact of the tissues involved. *pmr5* would not have been identified by screening mobile silencing impairment in roots, where the mutation had no discernible effects on symplasmic movement (Figure 3D–G). The tissue‐dependency and incompleteness of *pmr5's* effects on chlorosis suppression likely lie in extensive paralogy and genetic redundancy. Indeed, PMR5 belongs to a 46‐members protein family, while 27 PMR6‐related proteins exist in Arabidopsis (Vogel et al., 2002, 2004). Based on our data's interpretation, this genetic diversity suggests how various consortia of pectin‐modifying enzymes may alter PD frequency at some, unlike other cell‐to‐cell junctions, perhaps conditionally to local stress or developmental cues. The latter may include floral transition, which indeed correlates with increased secondary PD density in the SAM of mustard, which, like Arabidopsis, belongs to the Brassicaceae family (Ormenese et al., 2006). The spatio‐temporal fluidity thereby potentially granted to PD formation may underlie the poorly understood contextuality of si/miRNA‐ and, more generally, macromolecular movement (Voinnet, 2022). We note, finally, that *pmr5* impaired the CC → mesophyll translocation of amiRSUL in *pSUC2::amiRSUL* yet without detectable effect on amiRSUL movement from the CCs to systemic tissues, suggesting how the directionality of silencing movement might also be controlled, in addition to its contextuality.

## MATERIALS AND METHODS

### Plant material

All *Arabidopsis thaliana* plants used in this study were in the Col‐0 ecotype background. The following mutants were previously described: *dcl1‐11* (Zhang et al., 2008), *ago1‐42* (Poulsen et al., 2013), *hyl1‐2* (Vazquez et al., 2004), *dcl2* *‐1 dcl3* *‐1 dcl4‐2* (Fusaro et al., 2006), *pmr5* (here renamed *pmr5‐1*; Vogel et al., 2004), *pmr6‐3* (Vogel et al., 2002), *tbr‐1* (Potikha & Delmer, 1995), *rwa2‐3* (Manabe et al., 2011) and *hst‐1* (Telfer & Poethig, 1998). The following transgenic lines were previously described: *pSUC2::GFP*_*TM*_ (*pSUC2::*_*tm*_ *GFP9*; Stadler et al., 2005), *pSUC2::GFP*_*sol*_ (Imlau et al., 1999), *pSUC2::SUL:LUS* (*SUC‐SUL*; Xie et al., 2005), *CHS‐RNAi* (CHS‐ihpRNA; Wesley et al., 2001), *pSUC2::amiRSUL*, and *pSUC2::GUS* (Brioudes et al., 2021). Seeds from the *galt9‐3* T‐DNA insertion line (SALK_151601C) were obtained from the Eurasian Arabidopsis Stock Centre (uNASC). The PDLP3 reporter line was a kind gift from Dr. Andy Maule, formally group leader at the John Innes Center, UK. DNA sequencing of the fluorescent tag indicated that PDLP3 is fused to eGFP. This line, resistant to Basta herbicide, is therefore annotated as *pPDLP3::PDLP3:GFP*. Prior to characterization, *pmr5‐2* was backcrossed to the parental line *pATML1::amiRSUL* twice, or to Col‐0 twice for the version without *pATML1::amiRSUL* transgene.

### Cloning procedures/genotyping

DNA cloning was done using Phusion High‐Fidelity DNA Polymerase for PCR amplifications and the Gateway cloning technology (Thermo Fisher Scientific, Waltham, MA, USA). *CA1* (AT3G01500), *ATML1* (AT4G21750) and *PMR5* (AT5G58600) promoter sequences, as well as the *PMR5* genomic sequence, were PCR‐amplified from *Arabidopsis thaliana* genomic DNA using primers listed in Table S1. After purification on agarose gel, attB‐flanked DNA fragments were inserted via BP recombination into the pDONR P4‐P1r donor vector for promoters or into the pDONR221 for *PMR5*, giving rise to the entry vectors pENTR_attL4‐*pCA1*‐attR1, pENTR_attL4‐*pATML1*‐attR1, pENTR_attL4‐*pPMR5*‐attR1, and pENTR_attL1‐*PMR5*‐attL2. pENTR_attL1‐*PMR5* ^*S142A*^‐attL2, pENTR_attL1‐*PMR5* ^*D379A*^‐attL2 and pENTR_attL1‐*PMR5* ^*H382A*^‐attL2 mutant entry vectors were obtained by PCR‐based site‐directed mutagenesis of pENTR_attL1‐*PMR5*‐attL2 using primers listed in Table S1. The *pPMR5::PMR5:eGFP*, *pPMR5::PMR5* ^*S142A*^ *:eGFP*, *pPMR5::PMR5* ^*D379A*^ *:eGFP* and *pPMR5::PMR5* ^*H382A*^ *:eGFP* constructs were obtained by shuttling the *pPMR5* promoter sequences with the *PMR5*, *PMR5* ^*S142A*^, *PMR5* ^*D379A*^ or *PMR5* ^*H382A*^ sequences, together with the *eGFP* coding sequence cloned in an attR2‐attL3 entry vector, into the binary vector *pG7m34GW*, a vector derived from pP7m34GW (Karimi et al., 2007), in which a gentamicin‐resistance cassette had been inserted.

The *pATML1::amiRSUL* constructs were obtained by recombining the pENTR_attL4‐*pATML1*‐attR1 vector with attL1‐attL2 entry vectors that contain the *MIR319a* or *MIR390a* backbone modified to produce the miRNA UUAAGUGUCACGGAAAUCCCU targeting the *SUL* homolog *CH42* (AT4G18480) (de Felippes et al., 2011) into the binary vector *pB7m24GW* (Karimi et al., 2007). The *pCA1::P19* ^*WT*^ and *pCA1::P19* ^*WG*^ constructs were obtained by recombining the pENTR_attL4‐*pCA1*‐attR1 vector, respectively, with the pENTR_attL1*‐FHA‐P19*‐attL2 or the pENTR_attL1‐*FHA‐P19* ^*W39/42G*^‐attL2 (Brioudes et al., 2022), into the binary destination vector *pK7m24GW* (Karimi et al., 2007). The *pCA1::GUS and pATML1::GUS* constructs were obtained by recombining pENTR_attL4‐*pCA1*‐attR1 or pENTR_attL4‐*pATML11*‐attR1, respectively, with an attL1‐attL2 entry vector containing a 2xFLAG‐2xHA epitope tag coding sequence together with an attR2‐attL3 entry vector containing the β‐glucuronidase (GUS) coding sequence into the binary vector *pB7m34GW* (Karimi et al., 2007). The *pATML1::GFP*_*sol*_ construct was obtained by recombining pENTR_attL4‐*pATML1*‐attR1 with an attL1‐attL2 entry vector containing the *eGFP* coding sequence into the binary vector *pK7m24GW* (Karimi et al., 2007). The *pATML1::GFP*_*ER*_ vector was generated similarly, except that the *eGFP* coding sequence was fused to the coding sequences of the basic chitinase B signal peptide in 5′ and of a KDEL ER retention signal in 3′.

All binary vectors were introduced into *Agrobacterium* strain GV3101 to transform WT or mutant Arabidopsis plants by the floral dip method (Clough & Bent, 1998). T1 primary transformants were selected on MS plates containing appropriate selection. T2 plants were assessed for single‐locus insertions (3:1 segregation ratio) before propagation to homozygous T3 generations. Using Southern blot analysis and TAIL‐PCR (Liu et al., 1995), the *pATML1::amiRSUL* ^*319a*^ and *pATML1::amiRSUL* ^*390a*^ transgenes were located in the coding sequence of AT1G65950 and in the intergenic region between AT1G73660 and AT1G73670, respectively. Primers for genotyping these two transgenic lines are listed in Table S1.

### Growth conditions

Surface‐sterilized seeds were sown on MS medium containing MES buffer and vitamins (Duchefa Biochemie B. V, Haarlem, The Netherlands), supplemented with 1% sucrose (unless indicated otherwise) and solidified with 0.8% microagar. Plants were cultivated *in vitro* at 21°C in 12‐h light/12‐h dark conditions for 2 weeks before transplanting in soil, or at 22°C in 16‐h light/8‐h dark conditions for 10 days for the cell wall isolation used for the atomic force microscopy experiments. Subsequent growth was conducted at 21°C under 16‐h light/8‐h dark conditions, or in 12‐h light/12‐h dark for TuMV‐GFP infections. Light intensity was 120 μE m^−2^ sec^−1^ in every condition. Plant phenotype pictures were taken from 4‐ to 5‐week‐old plants, except for *in vitro* cultured plants, for which phenotyping pictures of 2‐week‐old plants were taken using a M205 FCA fluorescence stereo microscope (Leica, Wetzlar, Germany).

### Transient transformation in *Nicotiana benthamiana*

For agro‐infiltrations, leaves were infiltrated with equal volumes of agrobacterial suspensions in 10 mm MgCl_2_, 10 mm MES pH 5.6, and 200 μm acetosyringone. Final OD_600_ values were adjusted to 0.3. Fluorescent signals in agro‐infiltrated leaves were imaged by confocal at 3 days post‐infiltration.

### EMS mutagenesis and mutation mapping

The *pATML1::amiRSUL* ^*390a*^ reporter line was mutagenized as previously described for the *pSUC2::amiRSUL* reporter in Brioudes et al. (2021), except that mutants in which the *pATML1::amiRSUL* leaf chlorosis phenotype was reduced were screened among 1500 M2 progenies. Potential candidates were backcrossed once to the parental reporter *pATML1::amiRSUL*. One hundred F2 segregating mutant plants (2‐weeks‐old) were then harvested and pooled for genomic DNA extraction via CTAB (Clarke, 2009), in parallel with the *pATML1::amiRSUL* parental line. Whole‐genome resequencing of parental and pooled mutant DNA was performed using the TruSeq Nano DNA Library Prep kit and a HiSeq4000 sequencing system (Illumina, San Diego, CA, USA) to get an average genome coverage of 50×. Mutant DNA SNPs were mapped onto the Arabidopsis genome and filtered against the parental DNA SNPs using the CLC Genomics Workbench (Qiagen, Hilden, Germany) resequencing tools. Putative causal mutations were restricted to EMS transitions found in 100% of the sequencing reads and inducing amino acid changes or splice site defects in coding sequences.

### Arabidopsis micrografting procedure

Micrografting was done as described in Brioudes et al. (2021). Grafted plants were transferred to ½ MS medium without sucrose and grown for 3–4 weeks before harvesting, after the removal of plants with visible adventitious roots.

### Enrichment of epidermal tissues from Arabidopsis leaves using Meselect

Meselect (Svozil et al., 2016) was carried out mainly as described in (Brioudes et al., 2021). After harvesting the vasculature, the protoplasting solution was centrifuged at 100 ***g*** for 4 min at 4°C. Protoplasts, corresponding to the mesophyll‐enriched fraction, were gently washed once in washing buffer (154 mm NaCl, 125 mm CaCl_2_, 5 mm KCl, 2 mm MES, pH 5.7). One milliliter TRI Reagent (Merck, Darmstadt, Germany) was directly mixed with the protoplasts for RNA extraction. For each sample, tissues of 20–30 leaves harvested from 10 rosettes were pooled before proceeding to RNA extraction.

### Quantification of rosette surface and leaf chlorosis

The rosette surface (plant aerial area) and the degree of chlorosis were measured on photographs taken with a Canon G12 camera (Canon, Tokyo, Japan), using Color Threshold and Integrated density measurement settings and functions from NIH ImageJ. The chlorosis degree represents the visible chlorotic plant aerial area divided by the total visible plant aerial area.

### Quantification of average CW length

For aniline blue staining experiments and quantification of PDLP3 signals, the average of CW length per analyzed image was calculated independently for each experiment and each genotype. The area and perimeter of a minimum of 50 cells per condition were measured using, after brightness and contrast adjustments, the Freehand selection tool, and the Area and Perimeter measurement functions from NIH ImageJ software (NIH, Bethesda, MD, USA). The average number of cells and length of cell wall per mm^2^ could then be deduced and used to normalize the number of aniline blue deposits or PDLP3 signals by image by 100 μm of CW.

### TuMV‐GFP infection

TuMV‐GFP was as described in Garcia et al. (2014). TuMV‐GFP saps were prepared from 10 days post‐inoculation (dpi) infected Arabidopsis leaves. Infected tissues were ground with liquid nitrogen, resuspended in 1 × PBS (1 g tissue/2 ml PBS), and the resulting sap was diluted and used to inoculate 4‐week‐old Arabidopsis rosettes by gentle rubbing on cellite–dusted leaves. Three leaves per plant were inoculated and the total number of infection foci per plant was counted 5 dpi under epi‐fluorescent light. Leaf pictures were taken at 5 dpi under a UV lamp. Aerial tissues were collected individually at 6 dpi for molecular analyses.

### Immunoprecipitations (IPs)

For AGO1 and P19‐HA IPs, 4‐weeks‐old rosette leaves were ground in liquid nitrogen and resuspended in 3 ml for 1 g of tissue powder in IP buffer (50 mm Tris–HCl pH 7.5, 150 mm NaCl, 10% glycerol, 0.1% NP‐40), containing 2 μm MG‐132 and one tablet of cOmplete® protease inhibitor cocktail (Merck Roche, Basel, Switzerland) per 10 ml. All further steps were carried out on ice or in a 4°C cold chamber. After 30 min of gentle mixing, lysates were cleared from cell debris twice by centrifugation at 16 000 ***g*** for 10 min. Thirty microliters of cleared supernatants was mixed with 10 μl 4 × Western blot loading buffer (10% glycerol, 4% SDS, 62.5 mm Tris–HCl pH 6,8, 5% 2‐mercaptoethanol) for further analysis of input protein fractions. In addition, 100 μl was collected for input RNA extraction using 1 ml TRI Reagent (Merck). And 1 ml of cleared lysates was used for AGO1‐ or P19‐HA experiments. For AGO1 IPs, lysates were first pre‐cleared with 40 μl of protein A agarose beads (Merck Roche) for 30 min on a rotating wheel. Pre‐cleared lysates were then incubated with 1.25 μl of anti‐AGO1 antibody (Agrisera, Vannas, Sweden; ref. AS09 527) for 1 h under gentle mixing, followed by the addition of 40 μl of protein A agarose beads and another incubation for 1 h with gentle agitation. For P19‐HA IPs, lysates were incubated for 1 h on a rotating wheel with 30 μl of Anti‐HA magnetic beads (Pierce™, Thermo Fisher Scientific). Agarose or magnetic bead conjugates were washed three times with IP buffer and resuspended in 1 ml IP buffer. Bead conjugates collected from 100 to 200 μl of the last wash step were resuspended in 40 μL 1 × Western blot loading buffer for analysis of immunoprecipitated proteins. RNA was extracted from the remaining bead conjugates using the TRI Reagent protocol for analysis of immunoprecipitated RNA.

### Protein extraction and western analysis

For TuMV‐GFP analyses, total proteins were isolated from mock/infected 5‐week‐old individual rosettes. Plant tissues were ground in liquid nitrogen, and around 100 mg were resuspended in 1 ml TRI Reagent (Merck). About 200 μl chloroform was added, and tubes were shaken vigorously for 15 sec before being centrifuged for 15 min at 12 000 ***g*** at 4°C. Total proteins were precipitated overnight at −20°C from 200 μl of the phenol phase after adding 1 ml 0.1 m ammonium acetate in 100% methanol, washed twice with the same precipitating solution, and resuspended in 50 μl 3% SDS, 62.3 mm Tris–HCl pH 8, 10% glycerol buffer. Except for immunoprecipitation experiments, total plant proteins were isolated from leaves of 4–5‐week‐old rosettes by phenol extraction as described in (Schuster & Davies, 1983), with some modifications. Plant tissues ground in liquid nitrogen were resuspended in 0.7 m sucrose, 0.5 m Tris–HCl pH 8, 5 mm EDTA, 0.1 m NaCl, 2% 2‐mercaptoethanol, and cOmplete^®^protease inhibitor cocktail (Merck Roche, one tablet per 10 ml). One volume of Roti^®^Phenol (Carl Roth, Karlsruhe, Germany) was added, and the mixture shaken for 10 min at room temperature. The phenol phase was recovered after centrifugation at 16 000 ***g*** for 10 min at 4°C, and proteins were precipitated by the addition of five volumes of 0.1 m ammonium acetate in methanol, followed by 1 h of incubation at −20°C. Proteins were pelleted by centrifugation at 16 000 ***g*** for 20 min at 4°C and washed twice with 0.1 m ammonium acetate in methanol before resuspension in 3% SDS, 62.3 mm Tris–HCl pH 8, 10% glycerol. Protein concentrations were normalized using a modified Lowry procedure with the DC™ Protein Assay Kit (Bio‐Rad, Hercules, CA, USA), resolved on SDS‐PAGE gels, and electro‐transferred to Immobilon‐P PVDF membranes (Merck Millipore, Burlington, MA, USA). For AGO1 and GFP western analyses, after blocking for 30 min in 1 × TBS supplemented with 5% skim milk powder, anti‐AGO1 and anti‐GFP (Chromotek, Proteintech, Planegg‐Martinsried, Germany; ref. 3H9) antibodies were added to 1/8000 or 1/5000, respectively, and membranes incubated overnight at 4°C. Membranes were washed four times in 1 × TBS + 0.1% Tween‐20 and then incubated for 1 h at room temperature with horseradish peroxidase‐conjugated goat anti‐rabbit (for AGO1; Abcam, Cambrides, UK, ref. ab6721) or goat anti‐rat (for GFP; Cell Signaling Technology, Danvers, MA, USA, ref. 7077S) secondary antibodies. For FLAG western analyses, HRP‐conjugated anti‐FLAG (Sigma‐Aldrich, Merck; ref. A8592‐1MG) antibody was diluted to 1/5000 in the blocking solution and incubated for 1 h at room temperature. After washing four times in 1 × TBS + 0.1% Tween‐20, detection was performed using the ECL Western Blotting Detection Kit (GE HealthCare, Chicago, IL, USA) and revealed using the ChemiDoc™ Touch imaging system (Bio‐Rad). Membranes were stained with Coomassie blue to control total protein loading.

### RNA extraction and northern analysis

RNA was extracted from frozen tissues ground in liquid nitrogen using TRI Reagent (Merck) according to the manufacturer's instructions and resuspended in water. Equal amounts of RNA (1–10 μg depending on the experiment), dried with a vacuum concentrator, or immunoprecipitated RNA fractions were resuspended in Northern blot loading buffer (50% formamide, 10% glycerol, 10 mm Tris pH 7.7, 1 mm EDTA, 0.01% bromophenol blue), resolved by electrophoresis on a denaturing polyacrylamide gel (0.5 × TBE, 17.5% acrylamide/bisacrylamide 19:1, 8 m urea), transferred on a Hybond‐NX Nylon membrane (Cytiva, Marlborough, MA, USA) in 0.5 × TBE, and cross‐linked using 1‐ethyl‐3‐(3‐dimethylaminopropyl)carbodiimide (EDC) according to Pall and Hamilton (2008) for 2 h at 60°C. Membranes were incubated in PerfectHyb Plus Hybridization buffer (Sigma‐Aldrich, Merck) at 42°C overnight with an oligonucleotide probe complementary to a specific miRNA sequence and 5′‐end‐labeled with [γ‐^32^P]ATP using T4 PNK (Thermo Fisher Scientific). Oligonucleotide probe sequences are listed in Table S1. Membranes were washed three times for 15 min with 2 × SSC, 2% SDS at 50°C and exposed overnight or several days to a storage phosphor screen, which was subsequently imaged on a Typhoon FLA9000 (GE Healthcare). Band quantifications were done using Image Lab software (Bio‐Rad) with auto‐contrasted images. For sequential hybridizations of probes, membranes were stripped with boiling 0.1% SDS three times for 15 min before re‐probing with labeled oligonucleotides as described above.

### RT–qPCR and stem‐loop RT–qPCR

For RT–PCR and RT–qPCR, 1–2 μg of total RNA was treated with 1 unit of DNase I (Thermo Fisher Scientific) for a minimum of 30 min at 37°C and reverse‐transcribed with the RevertAid First Strand cDNA Synthesis Kit (Thermo Fisher Scientific) using a poly‐dT primer, according to the manufacturer's instructions. Stem‐loop RT–qPCR was carried out essentially according to Varkonyi‐Gasic et al. (2007), using the RevertAid First Strand cDNA Synthesis kit and by multiplexing stem‐loop RT primers listed in Table S1. One microliter of cDNA was used in 10 μl PCRs containing KAPA SYBR FAST qPCR 2X master mix (Sigma‐Aldrich, Merck) and gene‐specific or miRNA‐specific primers (0.2 μm each) listed in Table S1. qPCRs were performed in triplicates in 384‐well plates using a LightCycler 480 System (Roche, Basel, Switzerland) and following the PCR program recommended with the KAPA SYBR FAST qPCR mix. In addition, a melting curve was performed to verify the specificity of each PCR amplification. Cp values (cycle values of the maximum second derivative of the amplification curves) were calculated for each PCR with the LightCycler 480 software. Relative expression values for each mRNA or miRNA were obtained by calculating 2^−ΔCp^, where ΔCp represents the difference between the Cp value of the analyzed RNA and (i) the Cp values of *snoR85* (AT1G09873) small nucleolar RNAs in stem‐loop RT–qPCR experiments or (ii) the mean of the Cp values of *RHIP1* (AT4G26410) and *YLS8* (AT5G08290) control mRNAs in RT–qPCR experiments. Relative expression values from independent distinct samples were normalized with the mean of the control condition values and further individually plotted on graphs, together with their mean and standard deviation (SD). Normality distribution and homoscedasticity of the expression values were tested with Shapiro–Wilk and Fisher tests, respectively. All statistical analyses, as indicated in figure captions, were carried out using GraphPad Prism software (GraphPad Software, LLC).

### Plant tissue staining procedures, CF diffusion assay, and microscopy

Confocal pictures were acquired using a Zeiss LSM 780 microscope controlled by the Zeiss Zen software, except for confocal microscopy of immunolabeled cell walls (see corresponding paragraph below). Confocal image adjustments, and when required, z‐stack projections were further carried out using NIH ImageJ software. GFP‐tagged protein localization in leaves of young rosettes or in roots was analyzed in 2‐weeks‐old seedlings grown *in vitro* on MS plates. 488 nm laser excitation was used for GFP imaging, together with 500–550 nm emission detection band. Auto‐fluorescent signals were imaged with 600–700 nm emission detection band. GFP intensities were calculated using the “Plot z‐axis profile” function of NIH ImageJ. Values were first corrected so the maximum intensity for each sample was 100 and all the plots were then centered at 0 μm to compare all samples. GFP integration values represent individual plot surfaces (relative GFP intensity μm^−1^). Ten samples were measured per genotype.

For confocal imaging of the transient transformation in *Nicotiana benthamiana* leaves, 488 nm laser excitation was used for GFP signals, together with a 495–565 nm emission detection band; 594 nm laser excitation was used for mCherry_ER_ signals, together with a 598–696 nm emission detection band.

Aniline blue staining of rosette leaves and callose deposits numbering was performed as described in (Zavaliev & Epel, 2015). Aniline blue stained tissues were imaged using 405 nm laser excitation together with a 425–543 nm emission detection band. Ten images were taken per leaf sample. Seven leaf samples were used per genotype to calculate the average number of aniline blue deposits per 100 μm of CW.

GFP‐tagged PDLP3 was imaged in rosette leaves using a 514 nm excitation laser together with a 518–589 nm emission detection band. The number of PDLP3 fluorescing spots was calculated using the same approach as for aniline blue deposits' calculation. The NIH ImageJ protocol from (Zavaliev & Epel, 2015) was adapted with the following analysis parameters: rolling ball radius: 30, radius ball: 3, radius: 10, size range of ROI objects: 0.5–5, range of circularity: 0.5–1. Three leaves of WT and four leaves of *pmr5‐1* in the *pPDLP3::PDLP3:GFP* background were imaged. Ten images per sample were analyzed.

CF diffusion measurements were performed on rosette leaves following the Drop‐And‐See protocol as described in Cui et al. (2015), except that both adaxial and abaxial sides were analyzed. Instead of determining the diameter of fluorescent areas, total fluorescent areas, determined from a greyscale threshold value, were measured using NIH ImageJ software. Relative CF diffusion in rosette leaves corresponds to the ratio of the abaxial versus the adaxial fluorescent area corrected to the WT control. 488 nm laser excitation was used for CF imaging, together with a 500–555 nm emission detection band. At least 20 images from 10 or more plants were taken per genotype.

Basic Fuchsin staining of Arabidopsis roots was performed on 2‐week‐old seedlings grown *in vitro* on ½ MS vertical plates. Roots were cleared in 1 m KOH for 6 h at 37°C, then stained with 0.04% basic fuchsin for 10 min under gentle agitation. Roots were destained overnight in 70% ethanol at RT and rehydrated in water before confocal imaging. 561 nm laser excitation was used for imaging of basic fuchsin stained tissues, together with a 600–700 nm emission detection band.

GUS staining and embeddings were performed on 2‐week‐old seedlings grown *in vitro* on MS plates. Seedlings were harvested and prefixed for 20 min in 90% acetone at room temperature. They were washed once with staining buffer containing 0.2% Triton X‐100, 50 mm phosphate buffer (pH 7.2), 2 mm (for WT, *pATML1::GUS* and *pSUC2::GUS*) or 10 mm (for *pCA1::GUS*) K_4_[Fe(CN)_6_]/K_3_[Fe(CN)_6_]. They were further vacuum infiltrated for 1 h at room temperature and incubated overnight at 37°C in fresh staining buffer containing 2 mm X‐Gluc. Seedlings were fixed with FAA (3.2% formaldehyde, 50% ethanol, 5% acetic acid) for 2 h at room temperature, dehydrated with a series of ethanol buffer until removal of chlorophyll, and stained with eosin. After incubation with a concentration series of histoclear, samples were embedded in paraplast, and 8 μm sections were produced using a Leica RM2155 microtome. Images of sections were taken on a Leica DM2500 microscope equipped with a Leica DFC7000 T camera.

### Scanning electron microscopy (SEM)

SEM images were acquired on 2‐week‐old seedlings grown in vitro on MS plates. Microwave‐processed fixation, substitution, and resin embedding were performed as described in (Kraner et al., 2017). Ultra‐thin sections were cut with a diamond knife on a Leica EM UC7 Ultramicrotome. The sections were transferred onto Si‐wafer chips, which were mounted to SEM stubs via silver paint. Predefined areas were scanned in a TFS Magellan 400 scanning electron microscope (Thermo Fisher Scientific). Images with 5 × 5 nm pixel size were acquired at 1.9 kV and 1.6 nA by backscatter electron detection at a working distance of 2.7 mm. Hundreds of neighboring images were stitched to a larger tile scan panorama of the area (TFS MAPS). Images have been contrast‐inverted.

### Cell wall isolation

Cell wall dissection was performed using a modified method from (Novakovic et al., 2023). Each slide was covered with 200 μl of 0.1% poly‐d‐lysine hydrobromide (Sigma‐Aldrich, merck) (PDL) dissolved in deionized water in the center of the slide in a space marked with a hydrophobic pen to prevent spilling of PDL. Slides were left for 30 min for PDL to polymerize and then washed with deionized water. Slides were then dried under the stream of compressed N2. And 10‐day‐old cotyledons were cut from seedlings and placed on PDL‐coated slides under a stereomicroscope. Cotyledons were held with sharp dissection tweezers (Dumont, Montignez, Switzerland ref. #5) on the side close to the petiole. A short incision perpendicular to the central vein of the cotyledon was made mid‐cotyledon using a microsurgery scalpel (Fine Science Tools, Foster City, CA, USA; 150 feather blade scalpel), which was then dragged with enough force to remove the upper epidermis and other tissues of the cotyledon, leaving only the lower epidermis attached to the glass slide surface via interactions with PDL. The rest of the undissected cotyledons was attached to the glass slide using a drop of red nail polish applied using a thin syringe needle. Dissected cotyledons were left 15–20 min for the nail polish to dry and subsequently washed with 200 μl of 1% SDS for 2 min. SDS was washed by pouring deionized water from the undissected cotyledon's side towards the edge of the exposed lower epidermis to prevent its curling. Slides were then left to dry overnight.

### Immunolocalization of isolated cotyledon cell walls

Isolated cell walls of 10‐day‐old cotyledons were immunolabeled using monoclonal antibodies specific for dimethylesterified homogalacturonan (LM19) and for methylesterified homogalacturonan (LM20) from the plant cell wall carbohydrate antibody collection at University of Leeds, Centre for Plant Science, UK. Isolated dry cell walls were blocked using 3% BSA dissolved in 1 × PBS for 1 h. Afterwards, samples were washed three times for 5 min by carefully pipetting 200 μl of 1 × PBS on the dissected cell walls (slowly and gently pipetting to avoid curling of the cell wall). After each wash and before the next, removal of old PBS was done by gently leaning the glass slide edge on a piece of paper cloth, allowing the PBS to flow onto the paper. Two hundred microliter of primary antibody (LM19 or LM20) diluted to 1/5 in 3% BSA in 1 × PBS was then added to samples and left at 4°C overnight for incubation. On the next morning, samples were washed for 5 min three times with 200 μl of 1 × PBS, with the same care as the first series of washes to prevent cell wall curling. Each cell wall sample was then covered with 55 μl of Alexa 488 anti‐rat secondary antibody, diluted to 1/100 in 3% BSA dissolved in 1 × PBS. Samples were kept in the dark for 90 min and then imaged under a laser scanning confocal microscope. Two types of controls were used: no primary antibody control, where 3% BSA in 1 × PBS was added instead of LM19 or LM20 antibodies, and no secondary antibody control, where Alexa 488 antibody was replaced by 3% BSA in 1 × PBS.

### Laser scanning confocal microscopy and image analysis of immunolabeled cell walls

Confocal pictures were acquired using a Zeiss LSM 880 microscope controlled by the Zeiss Zen software (Zeiss, Oberkochen, Germany). A 40× dipping lens objective W Plan‐Apochromat 40×/1.0 DIC VIS‐IR M27 was used to avoid using coverslips on dissected cell wall samples. The zoom factor was 0.6 to give the widest possible imaging field. A 488 nm laser excitation was used for imaging of Alexa 488, together with a 600–700 nm emission detection band. Transmitted yellow light was used concurrently to locate and focus regions of interest of the epidermal cell wall. Signal gain was set on WT, avoiding signal saturation. Confocal image analyses were further carried out using NIH ImageJ software. Image preprocessing included background subtraction (rolling ball radius was set to 15 pixels) followed by applying a Gaussian blur filter (sigma radius was set to 1.5). Signal thresholds were adjusted using the Threshold tool, and binary masks were made and analyzed using the Analyze Particle tool. Measurements were set to capture Area (area covered by fluorescent signal) and mean gray intensity. The Analyze Particles tool circularity index was set to 0, while particle size was set in the range 0–10 000 μm^2^. The results display was set to be Overlay Mask, with the option Display Results, Summarize, and Add to ROI Manager active. Resulting overlay masks of the fluorescent signal areas were overlayed onto the original fluorescent image using the Overlay From ROI Manager option. The Multimeasure option in the ROI Manager was applied to the overlayed original fluorescent image. Relative average signal intensity of each image was calculated by summarizing Area and Mean Gray values for the entire image and dividing the Mean Gray Value sum by the Area sum. The resulting value is the number of pixels.μm^2^, also called Arbitrary Unit (AU) relative measure of fluorescent signal intensity.

### Mechanical characterization of cotyledon epidermal cell wall monolayers using atomic force microscopy

Mechanical characterization of cell wall monolayers was performed with a Bruker FastScan atomic force microscope (AFM) (Bruker, Billerica, MA, USA). Slides were put on the AFM stage, and samples were rehydrated using deionized water (100–200 μl). Cell wall monolayers were first identified using the AFM optical camera. Cell wall monolayers were identified as transparent patches of thin material at the edge of the lower epidermis of dissected cotyledons. After identifying regions of interest under the optical camera, imaging was performed using the PeakForce Mapping Quantitative Nanomechanics (PFM QNM) mode in deionized water to determine the material height. Cell wall monolayer regions of the dissected cotyledon's abaxial epidermis had height of 0.5–1.5 μm. PFM QNM in deionized water was then performed on these regions of cotyledons' adaxial epidermis with Force Capture mode turned on, to obtain force data. Indentation force was set at 3.5 nN. Bruker's ScanAsyst Fluid+ cantilevers (nominal spring constant 0.7 N//m) were used for mechanical mapping of the cell wall monolayers. PFM QNM measurements were performed on cell wall regions of 1 × 1 μm, with imaging resolution of 512 × 512 pixels, PFM force capture resolution of 128 × 128 indentation points, with each indentation point generating one force‐displacement curve. Raw force‐displacement curves obtained using PFM QNM were imported into the NanoScope Analysis v. 1.90 software (Bruker). Each PFM QNM scan corresponded to one 1 × 1 μm region of one pavement cell. Nearly, 200–300 force‐displacement curves were randomly selected from each 128 × 128 PFM QNM scan. Cell walls of 30 pavement cells, from 12 plants for WT and 12 plants for *pmr5‐1* (one cotyledon from each plant), from three biological replicates were analyzed. Force‐displacement curves were processed by changing their baseline, indentation point and curve fit, to enable calculation of the Young's modulus (elastic modulus) value for each force‐displacement curve.

### Imaging cell walls by atomic force microscopy and cellulose microfibrils diameter's measurement

Cotyledons dissected as described above were placed under the Dimension 3100 atomic force microscope (Veeco, Plainview, NY, USA). Imaging was performed on dry samples in air, using tapping mode, cantilever NCR‐ARROW (NanoWorld, Neuchatel, Switzerland) with a nominal spring constant of 42 N/m, on 1 × 1 μm cell wall patches, with a scan resolution of 512 × 512 pixels. Raw image files were imported into NanoScope Analysis v. 1.90 software (Bruker). The cellulose microfibrils diameter was analyzed using the cross‐section function. Twenty to forty fibers were analyzed per cell wall image (2–3 images per cotyledon) from one cotyledon per plant (20 plants of WT and 16 of *pmr5‐1*), from three biological replicates.

### Small RNA sequencing and bioinformatic analyses

Twenty micrograms of total RNA extracted from the whole aerial part of three Arabidopsis rosettes (3‐week‐old) grown on soil with a 12 h/12 h photoperiod at 21°C were resolved on a denaturating urea TBE 0.5X 17.5% polyacrylamide gel, together with 250 ng of ZR small‐RNA ladder (Zymo Research, Irvine, CA, USA). 17‐ to 29 nucleotides‐long RNA molecules were extracted from the gel using the ZR small‐RNA PAGE Recovery Kit (Zymo Research) and eluted in 6 μl of elution buffer. Small RNA sequencing libraries were prepared and barcoded using the Small RNA‐Seq Library Prep Kit (Lexogen, Vienna, Austria) according to the manufacturer's instructions. Individual library concentrations were gel quantified before multiplexing. Pooled libraries were further size selected on an 8% polyacrylamide gel before sequencing on a NovaSeq 6000 system (Illumina). Data processing and differential expression analysis were performed using sRNAbench and sRNAde from the online sRNAtoolbox (https://arn.ugr.es/srnatoolbox/; Aparicio‐Puerta et al., 2022) as of 19.09.2023. Sequences were mapped using the *Arabidopsis thaliana* genome assembly TAIR10 and miRNA annotations from miRbase v22.1. Other parameters were left as default. Tables and scatterplot obtained with the DESeq method are presented in Table S2.

## AUTHOR CONTRIBUTIONS

Conceptualization: FJ, YBA, OV. Methodology: FJ, OV. Validation: FJ, FB, AI, OV. Formal analysis: FJ. Investigation: FJ, FB, LN, AI. Resource: FB. Visualization: FJ. Writing – original draft: FJ, OV. Writing – review and editing: FJ, FB, LN, YBA, OV. Funding acquisition: OV, YBA.

## CONFLICT OF INTEREST

Authors declare that they have no competing interests.

## Supporting information


**Figure S1.** *pATML1::amiRSUL* Arabidopsis plants report miRNA activity/movement in a dose‐dependent manner.
**Figure S2.** Additional controls for the analyses of short‐range amiRSUL movement.
**Figure S3.** *pmr5* is causative of the suppressed amiRSUL‐mediated chlorosis.
**Figure S4.** *pmr5* reduces silencing without altering amiRSUL biogenesis or intracellular activity.
**Figure S5.** *pmr5* reduces phloem unloading and cell‐to‐cell movement of free‐GFP in leaves.
**Figure S6.** Additional controls for the analysis of the *pmr5* effect on phloem unloading and cell‐to‐cell movement in leaves and roots.
**Figure S7.** Pectin modifications impact on sRNA‐mediated silencing.

**Table S1.** List and sequences of primers used in this study.
**Table S2.** Analyses of the mature miRNA average normalized read count obtain by sRNA sequencing from two biological replicates of 3 week‐old rosettes in the *pmr5‐2* or in the *pmr5‐1* backgrounds (both without the *pATML1::amiRSUL* transgene) as compared with the WT control.

## Data Availability

sRNA deep sequencing data are available on Sequence Read Archive (SRA): bioproject accession # PRJNA1079042 (https://www.ncbi.nlm.nih.gov/bioproject/PRJNA1079042). Every other raw data used in this study (including raw image files, qPCR data) has been deposited in Zenodo (www.zenodo.org) under https://doi.org/10.5281/zenodo.11048049.
