# Supplementary material for: A pectin acetyl‐transferase facilitates secondary plasmodesmata formation and RNA silencing movement between plant cells
Source: Plant J. 2025 May 12;122(3):e70194. doi: 10.1111/tpj.70194 (PMC12068369; doi:10.1111/tpj.70194)
Supplement: Supplementary file 1 — Figure S1. pATML1::amiRSUL Arabidopsis plants report miRNA activity/movement in a dose‐dependent manner. Figure S2. Additional controls for the analyses of short‐range amiRSUL movement. Figure S3. pmr5 is causative of the suppressed amiRSUL‐mediated chlorosis. Figure S4. pmr5 reduces silencing without altering amiRSUL biogenesis or intracellular activity. Figure S5. pmr5 reduces phloem unloading and cell‐to‐cell movement of free‐GFP in leaves. Figure S6. Additional controls for the analysis of the pmr5 effect on phloem unloading and cell‐to‐cell movement in leaves and roots. Figure S7. Pectin modifications impact on sRNA‐mediated silencing. [file TPJ-122-0-s001.pdf]

Supplementary Figures for

A pectin acetyl-transferase facilitates secondary plasmodesmata formation and RNA silencing movement between plant cells

F. Jay, F. Brioudes, L. Novaković, A. Imboden, Y. Benitez-Alfonso, and O. Voinnet

Corresponding author: voinneto@ethz.ch

The PDF file includes:

Supplementary Figures 1 to 7

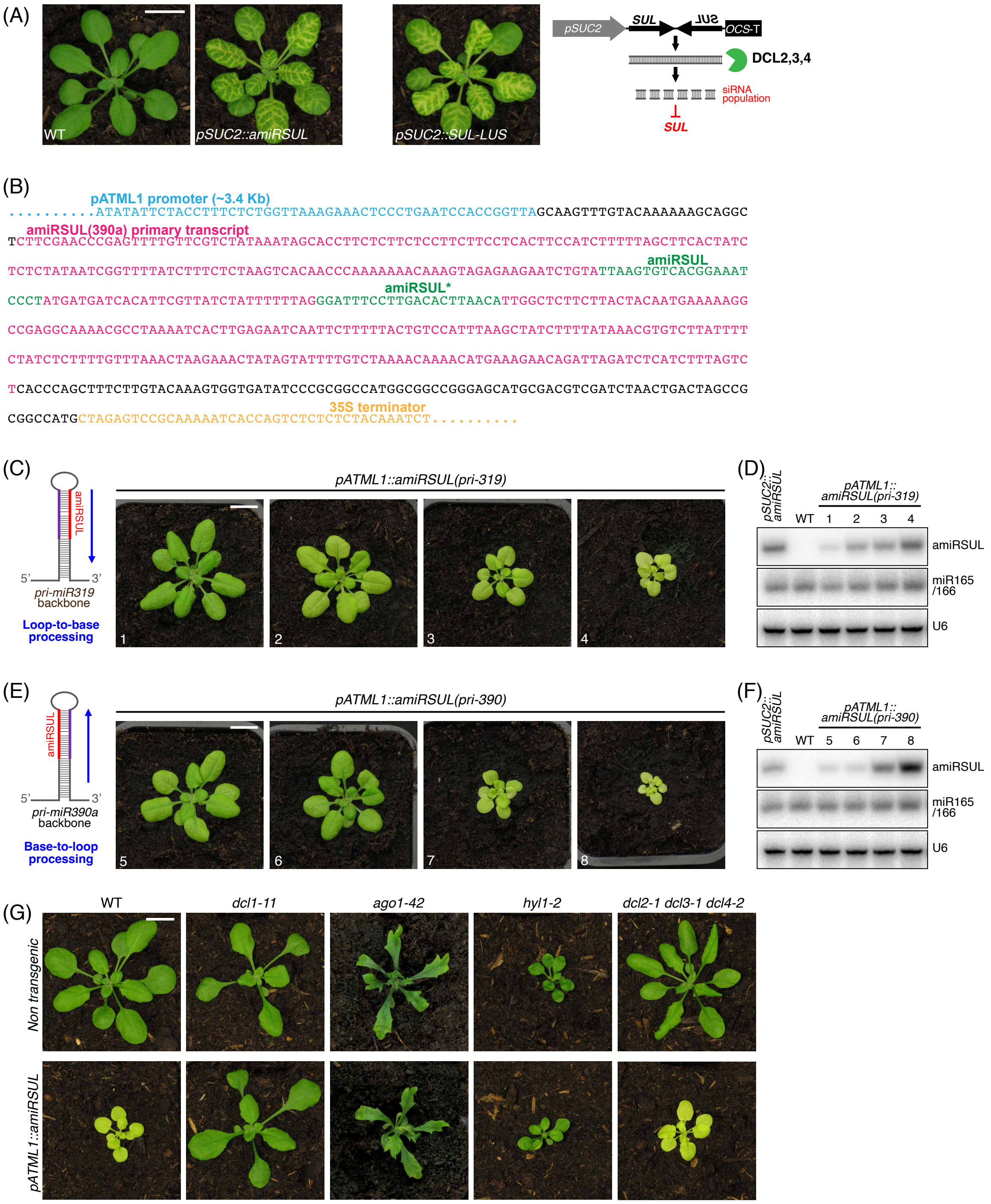

Sup Figure 1

20

Supplementary Figure 1. *pATML1::amiRSUL* Arabidopsis plants report miRNA activity/movement in a dose-dependent manner.

(A) Phenotype of *pSUC2::amiRSUL* and *pSUC2::SUL-LUS* rosettes as compared to WT. Scale bar: 1 cm. The scheme on the right depicts the principle of the siRNA-based *pSUC2::SUL-LUS* system. *OCS*-T: Octopine synthase terminator.

(B) Partial sequence of the *pATML1::amiRSUL* transgene carried by the parental line used in this study. The *pATML1* promoter's 3'-end is in blue, *amiRSUL* primary transcript in red, *amiRSUL* and *amiRSUL** in green, and the 35S terminator's 5'-end in yellow.

(C) Left: schematized *primary(pri)-miR319-based* amiRSUL construct. Right: Phenotype of *pATML1::amiRSUL(pri-319)* T1 rosettes with various degree of leaf chlorosis. Scale bar: 1 cm.

(D) amiRSUL northern analysis in the leaves of the plants depicted in (C), as compared to WT and *pSUC2::amiRSUL* depicted in (A). miR165/166 and U6 RNA hybridizations provide RNA loading controls.

(E) Left: schematized *primary(pri)-miR390a*-based amiRSUL construct. Right: phenotype of *pATML1::amiRSUL(pri-390a)* T1 rosettes with various degree of leaf chlorosis. Scale bar: 1 cm.

(F) Same as (D) for the plants depicted in (E).

(G) Phenotype of *dcl1-11*, *ago1-42*, *hyl1-2*, *dcl2-1 dcl3-1 dcl4-2* rosettes as compared to WT, without or with the *pATML1::amiRSUL* transgene. Scale bar: 1 cm. Chlorosis is reduced in mutants affecting the miRNA-, but not the siRNA- pathway.

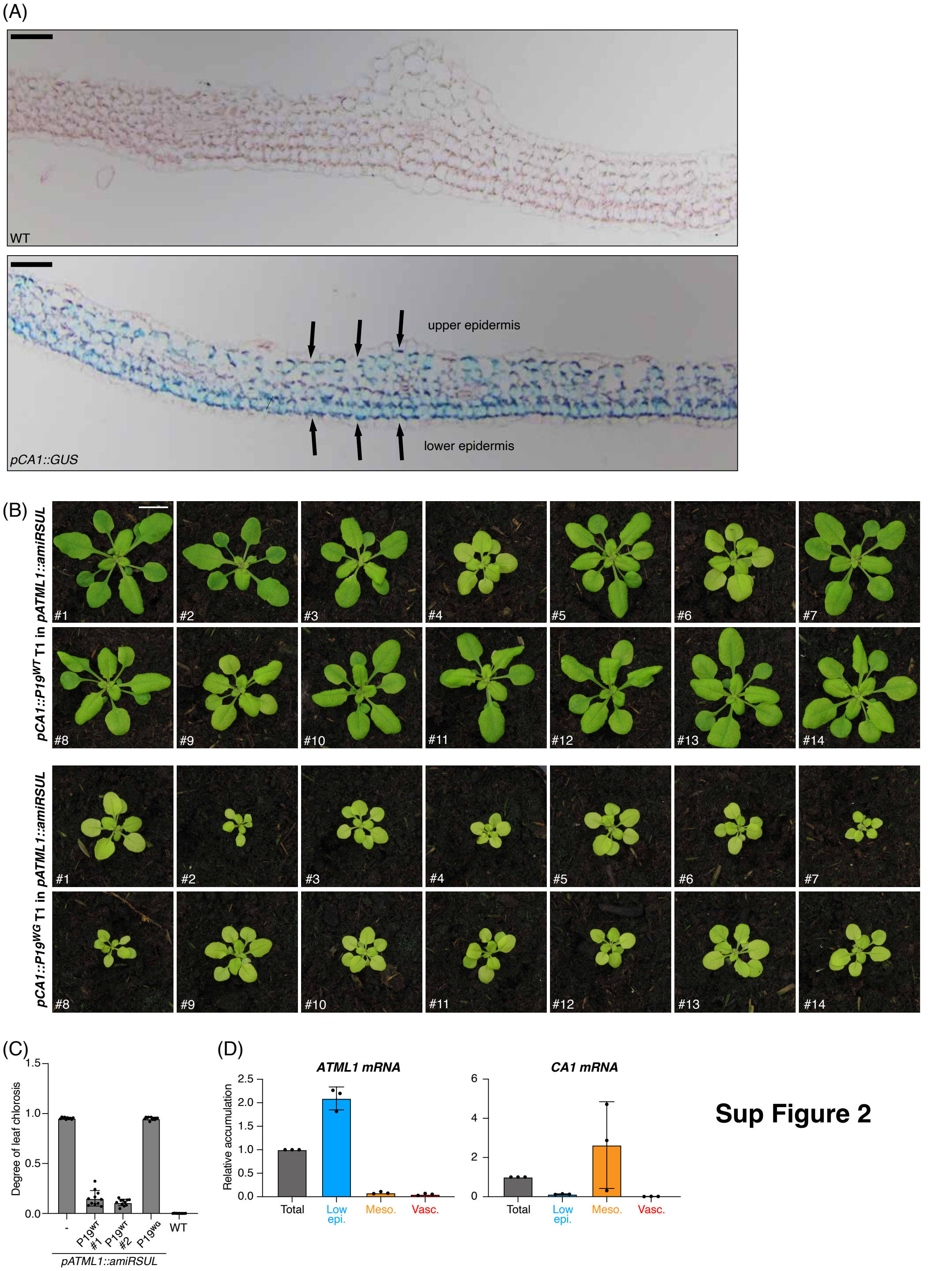

Sup Figure 2

Supplementary Figure 2. Additional controls for the analyses of short-range amiRSUL movement.

(A) GUS-staining pattern of WT (upper, no unspecific staining) or *pCA1::GUS* (bottom) leaf cross-sections demonstrating *pCA1* mesophyll-specific expression. Arrows indicate no GUS staining in the upper or lower epidermis. Scale bars: 10 μM. The image at the bottom is an uncropped view of that in Fig.1D.

(B) Range of phenotypes in *pCA1::P19*^{WT} (upper panel) and *pCA1::P19*^{WG} (lower panel) transgenic rosettes in individual T1s, all in the *pATML1::amiRSUL* background. Various degrees of leaf chlorosis recovery are consistently observed with the WT, but not with the dysfunctional WG version of P19. Scale bar: 1 cm.

(C) Quantification of the degree of leaf chlorosis of *pATML1::amiRSUL* rosettes without (-) or with the *pCA1::P19*^{WT} #1 (*P19*^{WT}#1), *pCA1::P19*^{WT} #2 (*P19*^{WT}#2) or *pCA1::P19*^{WG} (*P19*^{WG}) transgene, as compared to WT plants. Error bars: SD. $n \geq 10$.

(D) RT-qPCR analysis of *ATML1* (left) or *CA1* (right) accumulation within the epidermis and mesophyll enriched tissues, respectively, from *pATML1::amiRSUL* rosette leaves, validating the MeSelect procedure used for Fig.1F. Error bars: SD. $n = 3$.

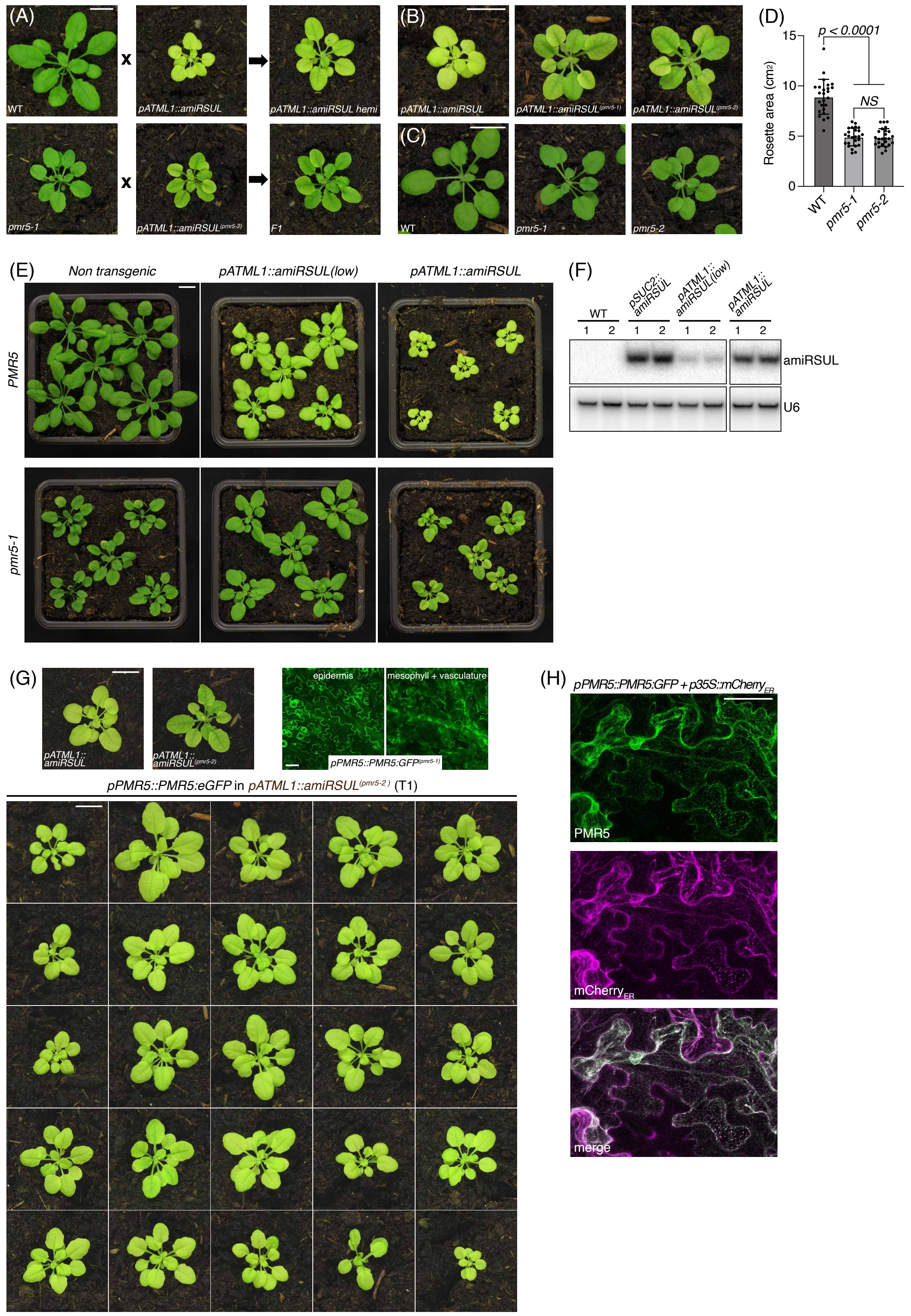

Sup Figure 3

Supplementary Figure 3. *pmr5* is causative of the suppressed amiRSUL-mediated chlorosis.

(A) Rosettes' phenotypes in the indicated genotypes used for allelism tests. Scale bar: 1 cm. The *pmr5-1* mutant phenotype, characterized by smaller rosettes and flat leaves is maintained in *pmr5-1 pmr5-2* F1 plants, which are less chlorotic as compared to *pATML1::amiRSUL* hemizygote (hemi) plants used as a reference.

(B) Rosettes' phenotypes in the indicated genotypes. Scale bar: 1 cm. *pmr5-1* reduces leaf chlorosis from *pATML1::amiRSUL*, as does the new *pmr5-2* allele also shown in Fig.2B.

(C) Phenotype of *pmr5-2* rosettes without the *pATML1::amiRSUL* transgene as compared with WT and *pmr5-1* rosettes. Scale bar: 1 cm. *pmr5-1* and *pmr5-2* have highly similar developmental phenotypes visible as reduced rosette size and flat leaves, as seen in (A).

(D) Surface quantification of four-week-old rosettes as depicted in (C). Error bars: SD. n = 25. Unpaired two-tailed t-test *p*-values are indicated.

(E) Phenotype of WT versus *pmr5-1* rosettes either without transgene or introgressed in the *pATML1::amiRSUL(low)* line, expressing low levels of amiRSUL, as compared to the *pATML1::amiRSUL* parental line used in this study. Scale bar: 1 cm.

(F) amiRSUL northern analysis in WT, *pSUC2::amiRSUL* or *pATML1::amiRSUL* rosette leaves, depicted in (E), in biological duplicates. U6 hybridization provides an internal RNA loading control. Despite showing developmental defects on its own (rosette size reduction, flatter leaves), the *pmr5-1* mutation allows correction of both chlorosis and size reduction induced by the *pATML1::amiRSUL* expression (E), independently of its expression level (F). This suggests that *pmr5-1* growth defects *per se* are not responsible for *pmr5-1* effect on amiRSUL-mediated chlorosis.

(G) Phenotype of individual *pATML1::amiRSUL*$^{(pmr5\text{-}2)}$ T1 rosettes with chlorosis complemented by *pPMR5::PMR5:eGFP*, as compared to controls. Scale bars: 1 cm. Upper right: confocal images of *pPMR5::PMR5:GFP* showing a GFP signal in the epidermis, mesophyll and vasculature of rosette leaves. Scale bar: 50 µM.

(H) Confocal images of *pPMR5::PMR5:GFP* and *p35S::mCherry*$_{ER}$ expression 3 days after transformation of *Nicotiana benthamiana* leaves via Agrobacterium infiltration. Upper panel: GFP channel, middle panel: mCherry channel, bottom panel: merged channels. Scale bar: 50 µm. The merged picture shows a high degree of colocalization (white signal), supporting ER localization of PMR5:GFP.

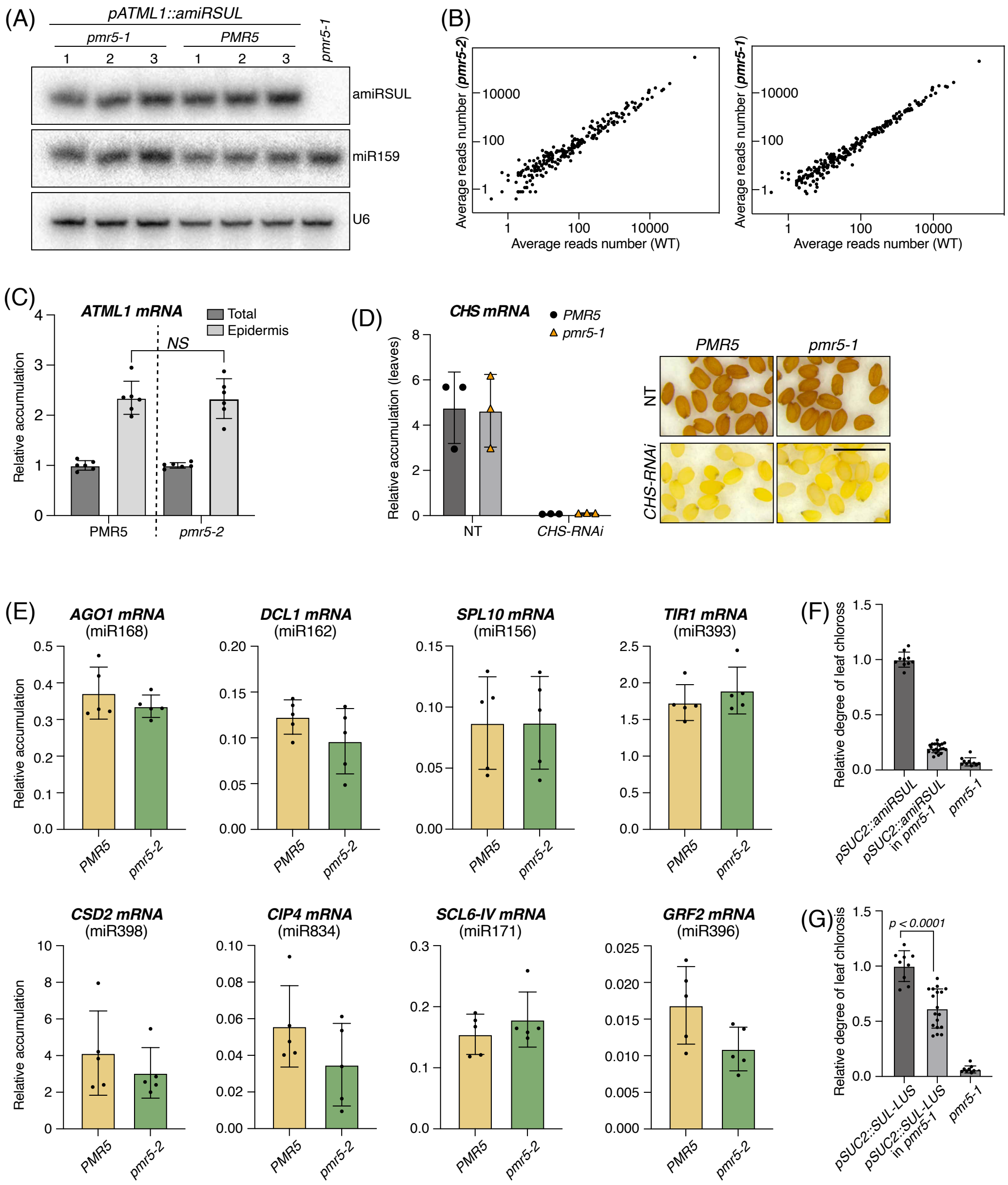

Sup Figure 4

Supplementary Figure 4. *pmr5* reduces silencing without altering amiRSULbiogenesis or intra-cellular activity.

(A) amiRSUL and miR159 northern analysis from rosette leaves of *pATML1::amiRSUL* in *pmr5-1* versus WT, in biological triplicates, as compared to *pmr5-1* without the *pATML1::amiRSUL* transgene. U6 hybridization provides an internal RNA loading control.

(B) Scatterplot representation of the mature miRNA average normalized read count obtain by sRNA sequencing from 2 biological replicates of 3 week-old rosettes in the *pmr5-2* background (left) or in the *pmr5-1* background (right) (both without the *pATML1::amiRSUL* transgene) as compared to the WT control.

(C) RT-qPCR of *ATML1* accumulation in total or epidermis-enriched leaf tissues from *PMR5* or *pmr5-2* in the *pATML1::amiRSUL* background. Error bars: SD. Unpaired two-tailed t-test result is indicated. *NS*: non-significant difference. n = 6.

(D) Left: RT-qPCR analysis of *CHS* accumulation in PMR5 or *pmr5-1* rosette leaves containing the *35S::CHS-RNAi* (*CHS-RNAi)* transgene or not (NT). Error bars: SD. n = 3. Right: pictures of seeds of the genotypes analyzed by RT-qPCR. Scale bar: 1 mm. *CHS* accounts for the seed coat's brown color in non-transgenic (NT) Arabidopsis.

(E) RT-qPCR analysis of the accumulation of various targets of endo-miRNAs not reported as mobile (*AGO1*, *DCL1*, *SPL10*, *TIR1*, *CSD2*, *CIP4*, *SCL6-IV* and *GRF2*) in *PMR5* or *pmr5-2* rosette leaves in the *pATML1::amiRSUL* background. Error bars: SD. n = 5.

(F) Relative leaf chlorosis quantification in the indicated genotypes. Error bars: SD. n ≥ 10.

(G) Relative leaf chlorosis quantification in the indicated genotypes. Error bars: SD. n ≥ 9. Unpaired two-tailed t-test *p*-value is indicated.

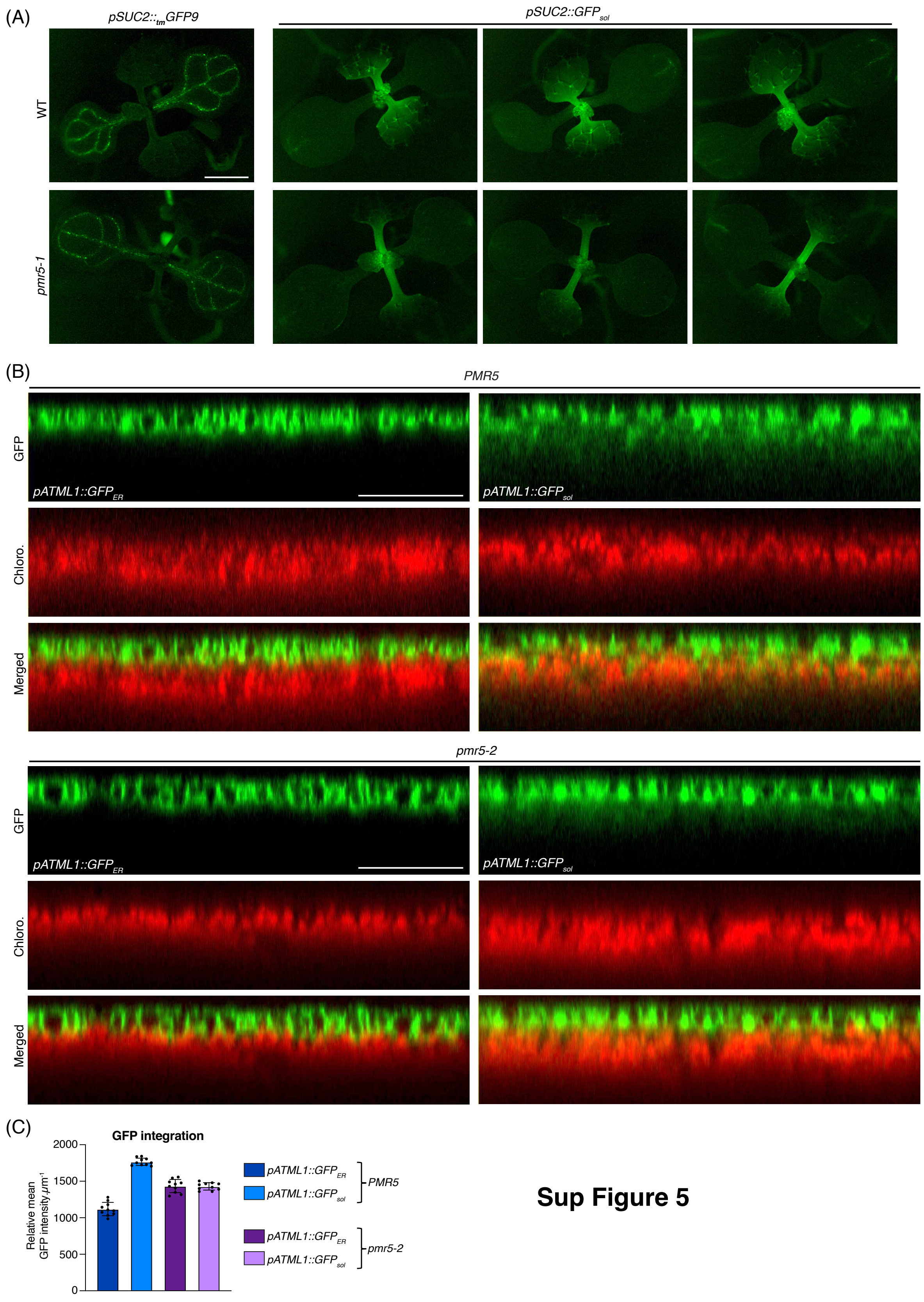

Sup Figure 5

Supplementary Figure 5. *pmr5* reduces phloem unloading and cell-to-cell movement of free-GFP in leaves.

(A) Representative phenotypes of *pSUC2::$_{tm}$GFP9* or *pSUC2::GFP$_{sol}$* seedlings in the *pmr5-1* or WT backgrounds observed under UV light.

(B) Orthogonal views of confocal stacks from the adaxial epidermis in young seedling leaves of *pATML1::GFP$_{ER}$* or *pATML1::GFP$_{sol}$* in *pmr5-2* or *PMR5*, in the *pATML1::amiRSUL* background. Chloro.: the auto-fluorescent red signal emitted between 600-700 nm allows approximate localization of the palisade mesophyll containing large amount of chlorophyll as opposed to the epidermis. Fig.3A is composed of crops from the images of the GFP channel shown here. Scale bars: 50 μM.

(C) Quantification of the integrated relative mean GFP intensities from the samples depicted in (B). Error bars: SD. n = 10. The integrated GFP intensity is higher in *pATML1::GFP$_{sol}$* than in *pATML1::GFP$_{ER}$* in young seedlings with the *PMR5* background. Together with those of Fig.3A, these results indicate that soluble GFP moves from the epidermis into the mesophyll. By contrast, no increase in the integrated intensity between *pATML1::GFP$_{ER}$* and *pATML1::GFP$_{sol}$* is observed in young seedlings with the *pmr5-2* mutant background, indicating that epidermis-to-mesophyll movement of soluble GFP is reduced in *pmr5-2* compared with WT.

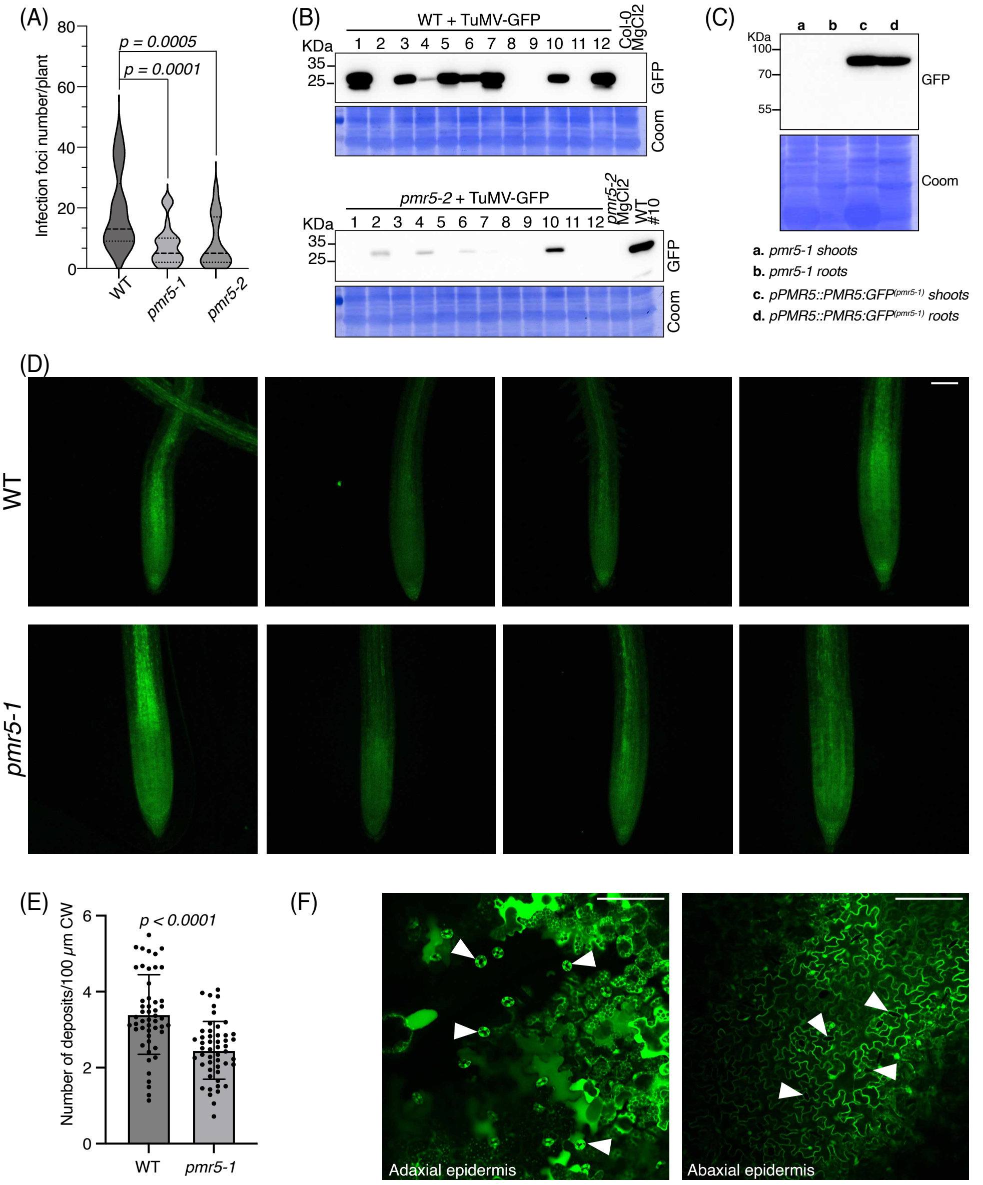

Sup Figure 6

Supplementary Figure 6: Additional controls for the analysis of the *pmr5* effect on phloem unloading and cell-to-cell movement in leaves and roots.

(A) Quantification of primary infection foci 5 days post-TuMV-GFP inoculation in the WT, *pmr5-1* or *pmr5-2* genotypes. Error bars: SD. Mann-Whitney two-tailed t-test *p* values are indicated. n = 25.

(B) GFP western analysis reporting TuMV-GFP accumulation in rosette leaves 7 days post-TuMV-GFP inoculation of WT or *pmr5-2* plants. Coomassie blue (Coom) staining provides a protein loading control. Comparable results were obtained for a second independent experiment.

(C) GFP-based western analysis of PMR5-GFP accumulation in shoots or roots of *pmr5-1* plants complemented (c, d) or not (a, b) with the *pPMR5::PMR5:GFP* genomic fusion. Coomassie blue (Coom) staining provides a protein loading control. Comparable results were obtained for one independent experiment.

(D) Additional confocal imaging of free GFP in roots of *pSUC2::GFP*$_{sol}$ plants with the indicated genotypes. Scale bar: 100 µm. While it shows plant-to-plant variation, GFP unloading in root tips displays the same pattern in the WT (upper panel) and *pmr5-1* (lower panel) backgrounds.

(E) Quantification of aniline blue deposits per 100 µm CW in WT or *pmr5-1* rosette leaves. Error bars: SD. Unpaired two-tailed t-test *p*-value is indicated. n = 50. Comparable results were obtained in one additional independent experiment.

(F) Confocal images of CFDA absorption/CF diffusion on the adaxial side (left panel) and diffusion on the abaxial side (right panel) of Arabidopsis rosette leaves. Scale bars: 100 µm. Arrows: stomata. Uptake of membrane-permeable CFDA and subsequent CF formation occurs in the adaxial epidermis, where it is applied, including in PD-deficient stomata guard cells. The specific absence of fluorescence in abaxial stomata guard cells supports symplastic movement of CF from the adaxial to the abaxial epidermis.

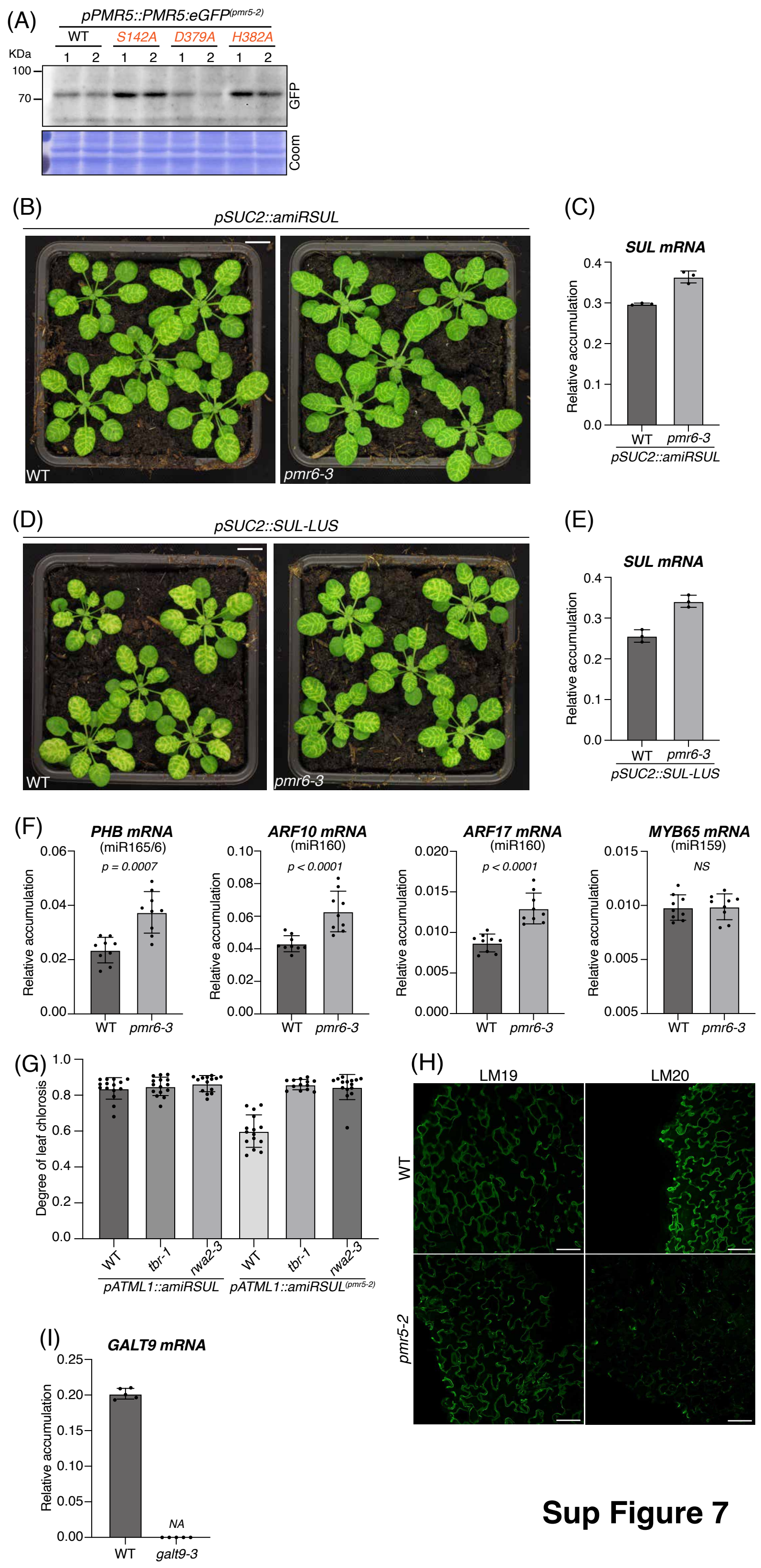

Sup Figure 7

Supplementary Figure 7. Pectin modifications impact on sRNA-mediated silencing. (A) GFP western analysis of PMR5-GFP accumulation in the plants depicted in

(Fig.4A), in biological duplicates. Coomassie blue (Coom) staining provides a protein loading control.

(B) Phenotype of WT or *pmr6-3* rosettes in the *pSUC2::amiRSUL* background. Scale bar: 1 cm.

(C) RT-qPCR analysis of *SUL* accumulation in rosette leaves depicted in (B). Error bars: SD. n = 3.

(D) Phenotype of WT or *pmr6-3* rosettes in the *pSUC2::SUL-LUS* background. Scale bar: 1 cm.

(E) RT-qPCR analysis of *SUL* accumulation in rosette leaves depicted in (D). Error bars: SD. n = 3.

(F) RT-qPCR analysis of *PHB*, *ARF10*, *ARF17* or *MYB65* accumulation in rosette leaves of *pmr6-3* or WT. Error bars: SD. Unpaired two-tailed t-test *p*-values are indicated. *NS*: non-significant difference. n = 9.

(G) Quantification of the degree of leaf chlorosis in *tbr-1, rwa2-3* or WT rosettes, in the *pATML1::amiRSUL* or *pATML1::amiRSUL*$^{(pmr5\text{-}2)}$ backgrounds. Error bars: SD. n ≥ 13.

(H) Confocal images of WT or *pmr5-2* cotyledon epidermal monolayers showing LM19 (left) or LM20 (right) signals. Scale bar: 50 µm.

(I) RT-qPCR analysis of *GALT9* accumulation in WT or *galt9-3* rosette leaves. Error bars: SD. n = 5. *NA*: no amplification. *GALT9* could not be amplified in the *galt9-3* T-DNA insertion, identifying it as a null mutant.
