# Supplementary material for: A pectin acetyl‐transferase facilitates secondary plasmodesmata formation and RNA silencing movement between plant cells
Source: Plant J. 2025 May 12;122(3):e70194. doi: 10.1111/tpj.70194 (PMC12068369; doi:10.1111/tpj.70194)
Supplement: Supplementary file 2 — Table S1. List and sequences of primers used in this study. Table S2. Analyses of the mature miRNA average normalized read count obtain by sRNA sequencing from two biological replicates of 3 week‐old rosettes in the pmr5‐2 or in the pmr5‐1 backgrounds (both without the pATML1::amiRSUL transgene) as compared with the WT control. [file TPJ-122-0-s002.pdf]

Supplementary Tables for

A pectin acetyl-transferase facilitates secondary plasmodesmata formation and RNA silencing movement between plant cells

F. Jay, F. Brioudes, L. Novaković, A. Imboden, Y. Benitez-Alfonso, and O. Voinnet

Corresponding author: voinneto@ethz.ch

The PDF file includes:

Supplementary Tables 1 and 2

Supplementary Table 1. List and sequences of primers used in this study.

Application	Primer name	Sequence	AGI locus identifier of amplified sequence or additional information
qRT-PCR	AGO1-qFor	AAGGAGGTCGAGGAGGGTATGG	AT1G48410
	AGO1-qRev	CAAATTGCTGAGCCAGAACAGTAGG	
	ARF10-qFor	TGGCGAGTCCATGTGTTATC	AT2G28350
	ARF10-qRev	CAACAAAGACGGAGATGGTG	
	ARF17-qFor	AGCACCTGATCCAAGTCCTTCTATG	AT1G77850
	ARF17-qRev	TGGTGAATAGCTGGGGAGGATTTC	
	ATML1-qFor	GAGGAGGAGGAGGTAGTGCT	AT4G21750
	ATML1-qRev	TGTGAGTAGTGAACCGCCAC	
	CA1-qFor	TCCGTAACGAGCCAGTTTTTT	AT3G01500
	CA1-qRev	CGGTTCCCATCTCTTCACTC	
	CHS-qFor	GACTGGAACTCCCTCTTCTGGA	AT5G13930
	CHS-qRev	CGCCCTCATCTTCTCTTCCTT	
	CIP4.1-qFor	CAGTGAGTTGACATCTACTCCAGTTAC	AT4G00930
	CIP4.1-qRev	CGTTCACAATTTCTCTTGAAGC	
	CSD2-qFor	TTTCATCTCCATGAGTTTGGTG	AT2G28190
	CSD2-qRev	AAAGGCTCTTCCAACAACAGA	
	DCL1-qFor	CAGAGTTCGCGATTCTTTTTG	AT1G01040
	DCL1-qRev	AGGGTTCAACATCAACATCCA	
	GALT9-For	TATCGAAGAGGAGTACAGTAAG	AT1G53290
	GALT9-Rev	TAGCAGAGAGAGTCGATCTG	
	GRF2-qFor	GCAGAACAGATGGGAAGAAAT	AT4G37740
	GRF2-qRev	TGTAGTAACCGCTTTGGATGC	
	MYB65-qFor	GATGGTTCCTGATAGCCATACAGTTAC	AT3G11440
	MYB65-qRev	TAGGCATCAACAGAGTCAAGGAGATC	
	NAC1-qFor	ACTTTGACCAAGAACCCTCTTCTTAT	AT1G56010
	NAC1-qRev	GAATCTTGAGTTCAGAGACTGAGTTG	
	PHB-qFor	AGAGTTCCTTTCCAAGGCTACAG	AT2G34710
	PHB-qRev	ATAGCGACTATGCCAATAGAATCC	
	RHIP1-qFor	GAGCTGAAGTGGCTTCAATGAC	AT4G26410
	RHIP1-qRev	GGTCCGACATACCCATGATCC	
	SCL6-IV-qFor	TGTCTAGCTCAGGGGATATTGG	AT4G00150
	SCL6-IV-qRev	AGCTGCTCTTTCTAATGGCTTC	
	SPL10-qFor	GTGGGAGAATGCTCAGGAGGC	AT1G27370
	SPL10-qRev	GAGTGTGTTTGATCCCTTGTGAATCC	
	SUL-qFor	GCAGGACAAGCTTCAAGACC	AT4G18480
	SUL-qRev	CGGTGCCTTAAGCAGTTAGG	
	TIR1-qFor	GCCTCTCTCTATCTGGCCTCTT	AT3G62980
	TIR1-qRev	AGGGCAGCTCTCTGGTCTCGA	
	YSL8-qFor	GAGTCTAGTGATTCTGGTCAG	AT5G08290
	YSL8-qRev	CACTGAATCATGTTCGAAGCAAGT	
Stem-loop qRT-PCR	amiRSUL-RTloop	GTCGTATCCAGTGCAGGGTCCGAGGTATTCGCACTGGATACGACAGGGAT	amiRSUL
	amiRSUL-For	CCGGCGTTAAGTGTCACGGAA	
	SnoR85-RTloop	GTCGTATCCAGTGCAGGGTCCGAGGTATTCGCACTGGATACGACACATGT	snoR85 - control
	SnoR85-For	CCGGCGGTGCATTCAAAAGCCCTT	
	Universal-Rev	AGTGCAGGGTCCGAGGTATTC	
Small RNA northern analysis	amiRSUL	AGGGATTTCCGTGACACTTAA	
	miR165/166	GGGGGATGAAGCCTGGTCCGA	
	U6	AGGGGCCATGCTAATCTTCTC	
	miR159	TAGAGCTCCCTTCAATCCAAA	
	mir160	TGGCATACAGGGAGCCAGGCA	
	miR390	GGCGCTATCCCTCCTGAGCTT	
Genotyping	pATML1-amiRSUL390-genomic-For	TGGTGACAGCCACACATTTT	Intergenic region between AT1G73660 and AT1G73670
	pATML1-amiRSUL390-genomic-Rev	TATTCGCCGTTTCTCTTGCT	
	pATML1-amiRSUL390-TDNA-LeftBorder	CAAAATATAGCGCGCAAACTAG	Use with pATML1-amiRSUL390-genomic-Rev primer
	pATML1-amiRSUL319-genomic-For	GTTGGATCGGCATTTTGTTT	AT1G65950
	pATML1-amiRSUL319-genomic-Rev	TCCACATCGTCAACCTGAAA	
	pATML1-amiRSUL319-TDNA-LeftBorder	TCGTTTCCCGCCTTCAGTTTA	Use with pATML1-amiRSUL319-genomic-Rev primer
	pmr5-2-For	CGCTGATCTCCTCATCTTCAACACTGGTCACTA	EMS, SpeI/BcuI cuts WT but not mutant
	pmr5-2-Rev	AGTCACTCGGGCTGATCAAT	
	pmr5-1-For	CCGTTCTTGGTGGACATAG	EMS, SmoI/SmlI cuts mutant but not WT (Chiniquy et al., 2019)
	pmr5-1-Rev	AGTCACTCGGGCTGATCAAT	
	galt9-3-For	AAGGATGTGATGGTTCTCGTG	SALK_151601C
	galt9-3-Rev	TCTGTGGAATCATTGCATCAC	
	pmr6-3-For	TGGAAATGTGGAGGTTAAGGAC	T-DNA insertion (Vogel et al., 2002)
	pmr6-3-Rev	GCATCGCTTCCAAATATCATCT	
	rwa2-3-For	TCGAATAGTCCACTCTGGTCC	SALK_013562
	rwa2-3-Rev	GCCTCTTGCATATTGTTCTTCC	
	tbr1-For	GCACGGTGGAGTTCTTTGTT	HpaII cuts WT but not mutant
	tbr1-For	CTTTGCCGGATTCACATTCT	
Cloning	PMR5-attB1-For	GGGGACAAGTTTGTAAAAAGCAGGCTTAAGAATGGGTTCTCTTCTTCCTCTTC	PMR5 genomic DNA
	PMR5-attB2-Rev	GGGGACTGCTTTTTAGAAAGCTGGGTTTCCATAGATGAGAAGCGTATACAAC	
	pPMR5-attB4-For	GGGGACAACTTTGTATAGAAAAGTTGATCAAGAGCGGCGTAGCAGATC	PMR5 promoter
	pPMR5-attB1r-Rev	GGGGACTGCTTTTTTGTACAAACTTGTCTTTTGCTTTTTCGTGGAAAAC	
	PMR5-S142A-mut-For	TGCGGGTGATGCATTGGGGAAGAATCAATGG	PMR5 catalytic mutants obtained by PCR-based site-directed mutagenesis
	PMR5-S142A-mut-Rev	TTCCCCAATGCATCACCCGCAAACATTATGG	
	PMR5-D379A-mut-For	CCAGTCTGCAGCTTGTAGCCATTGGTGTTTG	
	PMR5-D379A-mut-Rev	TGGCTACAAGCTGCAGACTGGTCTGGTCTAG	
	PMR5-H382A-mut-For	AGATTGTAGCGCTTGGTGTTTGCCTGGTTTAC	
	PMR5-H382A-mut-Rev	GCAAACACCAAGCGCTACAATCTGCAGACTG	
	pATML1-attB4-For	GGGGACAACTTTGTATAGAAAAGTTGCTATTGATTCTGAACTGTACCC	ATML1 promoter
	pATML1-attB1r-Rev	GGGGACTGCTTTTTTGTACAAACTTGCTAACCGGTGGATTCAGGGAG	
	pCA1-attB4-For	GGGGACAACTTTGTATAGAAAAGTTGCTGGATCCTTTAGCCTTGTTC	CA1 promoter
	pCA1-attbB1r-Rev	GGGGACTGCTTTTTTGTACAAACTTGCTTGTTGGCGAAGAGAAGCGG	

Supplementary Table 2. Analyses of the mature miRNA average normalized read count obtain by sRNA sequencing from 2 biological replicates of 3 week-old rosettes in the *pmr5-2* or in the *pmr5-1* backgrounds (both without the *pATML1::amiRSUL* transgene) as compared to the WT control.

Comparative analysis WT versus *pmr5-1*			Comparative analysis WT versus *pmr5-2*		
**Mature miRNA name**	**mean of normalized read count for WT**	**mean of normalized read count for *pmr5-1***	**Mature miRNA name**	**mean of normalized read count for *pmr5-2***	**mean of normalized read count for WT**
ath-miR841b-5p	58.48419927	10.556781	ath-miR860	0	9.336400016
ath-miR5656	4.920930278	0	ath-miR841b-5p	15.71889836	58.48419927
ath-miR844-5p	5.635001814	0.850981496	ath-miR844-5p	5.732603551	5.635001814
ath-miR157b-5p	24107.1781	17602.06083	ath-miR157b-5p	16636.67893	24107.1781
ath-miR5629	2.308023629	0	ath-miR5629	0	2.308023629
ath-miR166b-3p	9219.395422	13277.0038	ath-miR166b-3p	12201.77522	9219.395422
ath-miR865-5p	4.07581822	1.542320646	ath-miR865-5p	15.71889836	4.07581822
ath-miR167c-5p	14.63175031	14.1731191	ath-miR167c-5p	10.03779342	14.63175031
ath-miR169n	94.18501179	74.19103335	ath-miR169n	83.36316913	94.18501179
ath-miR169m	128.6315237	101.7931626	ath-miR169m	112.0776855	128.6315237
ath-miR169l	93.20082573	74.19103335	ath-miR169l	82.97412908	93.20082573
ath-miR169k	94.21978029	75.65353282	ath-miR169k	84.11549993	94.21978029
ath-miR169j	94.18501179	74.19103335	ath-miR169j	83.36316913	94.18501179
ath-miR390a-3p	38.66140416	45.81707722	ath-miR390a-3p	41.89037949	38.66140416
ath-miR169i	95.54361786	79.93482518	ath-miR169i	85.67166013	95.54361786
ath-miR169h	94.21978029	75.65353282	ath-miR169h	84.11549993	94.21978029
ath-miR169e	4.955698777	2.233659796	ath-miR169e	3.060821801	4.955698777
ath-miR169d	4.955698777	2.233659796	ath-miR169d	3.060821801	4.955698777
ath-miR169c	10.59070059	12.57802868	ath-miR169c	22.54137415	10.59070059
ath-miR472-5p	252.5426947	191.00763	ath-miR472-5p	138.4266112	252.5426947
ath-miR829-3p.1	6.31430485	14.22655539	ath-miR829-3p.1	11.98299367	6.31430485
ath-miR162a-5p	63.81431806	58.58046662	ath-miR162a-5p	71.04543451	63.81431806
ath-miR171a-3p	1653.842021	1821.882845	ath-miR171a-3p	1040.080411	1653.842021
ath-miR172b-5p	7.943025443	16.72606359	ath-miR172b-5p	12.68382586	7.943025443
ath-miR172c	5.225813297	8.21691515	ath-miR172c	9.571505454	5.225813297
ath-miR164b-5p	102.3714167	84.26822079	ath-miR164b-5p	71.32867685	102.3714167
ath-miR827	39.11339465	24.35717912	ath-miR827	22.98191281	39.11339465
ath-miR172a	14.1530248	21.99159491	ath-miR172a	31.77533816	14.1530248
ath-miR825	118.7361931	107.9076759	ath-miR825	86.55273745	118.7361931
ath-miR156b-5p	5568.244957	4822.994027	ath-miR156b-5p	3869.263754	5568.244957
ath-miR823	239.9355508	246.3972493	ath-miR823	383.9416889	239.9355508
ath-miR844-3p	2.03790911	5.823613008	ath-miR844-3p	17.12056274	2.03790911
ath-miR157b-3p	216.2829516	309.2893923	ath-miR157b-3p	456.1430416	216.2829516
ath-miR167c-3p	31.77210177	19.01315967	ath-miR167c-3p	23.13640863	31.77210177
ath-miR447b	46.20327456	28.02762003	ath-miR447b	38.38901903	46.20327456
ath-miR390b-5p	2224.119166	3198.420409	ath-miR390b-5p	2905.852237	2224.119166
ath-miR167d	543.2065645	433.4685901	ath-miR167d	407.673132	543.2065645
ath-miR777	5.704538812	14.7853036	ath-miR777	7.314513059	5.704538812
ath-miR167b	3633.567365	3713.869457	ath-miR167b	2810.019562	3633.567365
ath-miR472-3p	74.54409265	84.42786314	ath-miR472-3p	57.24318661	74.54409265
ath-miR775	352.8120642	322.0501156	ath-miR775	446.9717782	352.8120642
ath-miR162a-3p	657.3696421	708.1843597	ath-miR162a-3p	1415.64463	657.3696421
ath-miR171b-5p	972.3413137	1385.604794	ath-miR171b-5p	1707.765011	972.3413137
ath-miR5663-5p	43.17314592	37.86734351	ath-miR5663-5p	24.76981675	43.17314592
ath-miR824-5p	27.80058905	21.7257465	ath-miR824-5p	41.03505148	27.80058905
ath-miR172b-3p	13.13407024	21.99159491	ath-miR172b-3p	25.29040381	13.13407024
ath-miR170-5p	10.28581757	15.79526092	ath-miR170-5p	16.18518632	10.28581757
ath-miR164b-3p	25.38825992	12.68423474	ath-miR164b-3p	11.59395362	25.38825992
ath-miR447a-3p	46.20327456	28.02762003	ath-miR447a-3p	38.38901903	46.20327456
ath-miR5020b	36.38814903	17.44445414	ath-miR5020b	31.02300737	36.38814903
ath-miR833b	9.980934553	8.961690588	ath-miR833b	7.288763755	9.980934553
ath-miR156b-3p	77.0874623	89.95924286	ath-miR156b-3p	134.4102647	77.0874623
ath-miR166g	9250.079032	13310.82865	ath-miR166g	12217.51987	9250.079032
ath-miR166f	9252.116941	13312.71664	ath-miR166f	12223.66726	9252.116941
ath-miR166d	9217.017862	13276.23264	ath-miR166d	12195.65357	9217.017862
ath-miR166c	9226.38903	13290.0337	ath-miR166c	12201.82672	9226.38903
ath-miR157c-5p	36447.69996	27203.27253	ath-miR157c-5p	28979.73441	36447.69996
ath-miR402	19.31733457	22.25744331	ath-miR402	59.89482005	19.31733457
ath-miR3932b-5p	352.8146988	319.41735	ath-miR3932b-5p	229.8879745	352.8146988
ath-miR841a-5p	93.54851073	42.62556336	ath-miR841a-5p	65.49587658	93.54851073
ath-miR400	247.5202232	191.5122755	ath-miR400	181.4382133	247.5202232
ath-miR833a-5p	41.31711278	34.91396013	ath-miR833a-5p	32.73366339	41.31711278
ath-miR866-5p	2.377560628	2.818792889	ath-miR866-5p	5.3435635	2.377560628
ath-miR4228-5p	1.968372111	5.39812226	ath-miR4228-5p	3.838901903	1.968372111
ath-miR160a-5p	8.996748497	8.482763552	ath-miR160a-5p	9.623004061	8.996748497
ath-miR390b-3p	74.8757107	136.3066839	ath-miR390b-3p	131.2492462	74.8757107
ath-miR165b	9517.964655	16573.97242	ath-miR165b	14979.63305	9517.964655
ath-miR162b-5p	25.83221694	21.99092839	ath-miR162b-5p	17.74134653	25.83221694
ath-miR171b-3p	31.97267929	51.64069023	ath-miR171b-3p	41.2152966	31.97267929
ath-miR824-3p	278.5648211	286.8445887	ath-miR824-3p	191.6478503	278.5648211
ath-miR447a.2-3p	67.20016517	37.54672579	ath-miR447a.2-3p	105.9901927	67.20016517
ath-miR164c-5p	38.80047816	44.99248061	ath-miR164c-5p	29.64709229	38.80047816
ath-miR170-3p	233.3617995	218.077396	ath-miR170-3p	146.5449537	233.3617995
ath-miR156c-5p	5302.947881	4537.879785	ath-miR156c-5p	3507.314302	5302.947881
ath-miR164a	94.28931729	74.58880617	ath-miR164a	61.31663274	94.28931729
ath-miR157c-3p	2207.129075	1886.571436	ath-miR157c-3p	4378.984711	2207.129075
ath-miR5014b	0.679303037	3.084641292	ath-miR5014b	0.778080102	0.679303037
ath-miR3932b-3p	4.241627241	4.281292362	ath-miR3932b-3p	0.778080102	4.241627241
ath-miR841a-3p	391.6179412	245.6254225	ath-miR841a-3p	270.802681	391.6179412
ath-miR833a-3p	3.257441186	0.69133915	ath-miR833a-3p	6.951222311	3.257441186
ath-miR4228-3p	29.80372966	23.05498852	ath-miR4228-3p	16.26243423	29.80372966
ath-miR869.2	11.87976967	7.977451632	ath-miR869.2	5.006022056	11.87976967
ath-miR826a	2.987326666	0.850981496	ath-miR826a	1.167120153	2.987326666
ath-miR162b-3p	828.8239921	878.5566882	ath-miR162b-3p	1653.061514	828.8239921
ath-miR159c	522.8891066	559.5120592	ath-miR159c	742.4065831	522.8891066
ath-miR159a	21747.27456	18546.74656	ath-miR159a	14406.51917	21747.27456
ath-miR171c-5p	282.2742528	360.7400562	ath-miR171c-5p	333.7700697	282.2742528
ath-miR164c-3p	17.92396	22.01797979	ath-miR164c-3p	25.9626862	17.92396
ath-miR156c-3p	351.3758877	349.4147809	ath-miR156c-3p	327.9031186	351.3758877
ath-miR846-5p	16.5038504	16.99191199	ath-miR846-5p	19.94684031	16.5038504
ath-miR158b	475.257689	495.1864677	ath-miR158b	911.7532045	475.257689
ath-miR169f-5p	4.955698777	2.233659796	ath-miR169f-5p	3.060821801	4.955698777
ath-miR840-5p	99.59270106	90.17165498	ath-miR840-5p	110.8590667	99.59270106
ath-miR5654-5p	3.736166701	7.977451632	ath-miR5654-5p	7.288763755	3.736166701
ath-miR157d	978.5941151	616.4202803	ath-miR157d	534.2941942	978.5941151
ath-miR171c-3p	33.67093688	57.78358793	ath-miR171c-3p	46.94790015	33.67093688
ath-miR861-5p	1.968372111	0	ath-miR861-5p	4.565483398	1.968372111
ath-miR156d-5p	11578.36298	9662.23519	ath-miR156d-5p	7582.952252	11578.36298
ath-miR3932a	2.308023629	2.393302141	ath-miR3932a	0.778080102	2.308023629
ath-miR5645f	3.361746684	3.084641292	ath-miR5645f	1.167120153	3.361746684
ath-miR398a-5p	50.95036234	29.09101364	ath-miR398a-5p	23.34240306	50.95036234
ath-miR5645e	1.698257592	2.313480969	ath-miR5645e	0.389040051	1.698257592
ath-miR846-3p	21.5905897	17.28481179	ath-miR846-3p	13.85094602	21.5905897
ath-miR5645d	3.361746684	3.084641292	ath-miR5645d	1.167120153	3.361746684
ath-miR5645b	3.361746684	3.084641292	ath-miR5645b	1.167120153	3.361746684
ath-miR5645a	3.361746684	3.084641292	ath-miR5645a	1.167120153	3.361746684
ath-miR156j	38.18267865	22.76208871	ath-miR156j	40.77475794	38.18267865
ath-miR160b	6.619187869	6.328924929	ath-miR160b	6.147392906	6.619187869
ath-miR156h	103.8075933	103.3084319	ath-miR156h	102.7922229	103.8075933
ath-miR156g	45.30732705	34.2232875	ath-miR156g	37.76543475	45.30732705
ath-miR841b-3p	411.9542304	259.7985416	ath-miR841b-3p	285.7434992	411.9542304
ath-miR166e-5p	1210.559776	858.3191596	ath-miR166e-5p	663.9531294	1210.559776
ath-miR156e	5306.64928	4538.225455	ath-miR156e	3512.709364	5306.64928
ath-miR2933b	0	0.771160323	ath-miR830-5p	8.793425352	5.939884833
ath-miR2933a	0	0.771160323	ath-miR1886.2	14.94081826	17.9587285
ath-miR830-5p	5.939884833	6.940442906	ath-miR169f-3p	115.704992	109.9480556
ath-miR1886.2	17.9587285	13.13611037	ath-miR840-3p	35.56274146	22.98396427
ath-miR169f-3p	109.9480556	143.4879233	ath-miR5654-3p	46.37861498	33.81804436
ath-miR840-3p	22.98396427	16.32695772	ath-miR858b	25.80819038	18.15930602
ath-miR5654-3p	33.81804436	21.80490116	ath-miR858a	123.0766046	79.97048347
ath-miR858b	18.15930602	29.41029833	ath-miR861-3p	3.501360459	9.676051534
ath-miR858a	79.97048347	122.7200309	ath-miR172d-3p	9.571505454	5.225813297
ath-miR861-3p	9.676051534	6.355309813	ath-miR156d-3p	430.5665949	208.7651816
ath-miR172d-3p	5.225813297	8.21691515	ath-miR398a-3p	34.88765857	16.2604709
ath-miR156d-3p	208.7651816	208.7720354	ath-miR408-5p	941.6639356	803.2163235
ath-miR398a-3p	16.2604709	20.55548032	ath-miR5643b	6.147392906	4.616047259
ath-miR408-5p	803.2163235	1088.014731	ath-miR5643a	6.147392906	2.647675148
ath-miR5643b	4.616047259	4.653346822	ath-miR166e-3p	12222.88918	9252.116941
ath-miR5643a	2.647675148	3.616338097	ath-miR5998b	3.449861852	2.952558167
ath-miR166e-3p	9252.116941	13310.82865	ath-miR5998a	3.449861852	2.952558167
ath-miR5998b	2.952558167	1.887990221	ath-miR868-5p	7.288763755	7.194185408
ath-miR5998a	2.952558167	1.887990221	ath-miR779.2	244.8629436	194.6121568
ath-miR868-5p	7.194185408	4.89281034	ath-miR160c-5p	10.40108416	8.996748497
ath-miR779.2	194.6121568	192.0182539	ath-miR830-3p	0	2.03790911
ath-miR160c-5p	8.996748497	8.482763552	ath-miR8183	41.9418781	24.33453687
ath-miR830-3p	2.03790911	3.350489694	ath-miR169g-5p	3.060821801	4.955698777
ath-miR8183	24.33453687	21.21976806	ath-miR5642b	367.1273173	493.5159017

Comparative analysis WT versus *pmr5-1*			Comparative analysis WT versus *pmr5-2*		
**Mature miRNA name**	**mean of normalized read count for WT**	**mean of normalized read count for *pmr5-1***	**Mature miRNA name**	**mean of normalized read count for *pmr5-2***	**mean of normalized read count for WT**
ath-miR169g-5p	4.955698777	2.233659796	ath-miR5642a	654.8277636	871.4648128
ath-miR5642b	493.5159017	648.7134721	ath-miR8180	40.09967506	25.65837444
ath-miR5642a	871.4648128	1087.105923	ath-miR5027	0	6.209999352
ath-miR8180	25.65837444	42.49363893	ath-miR5026	727.1035637	1931.636666
ath-miR5027	6.209999352	1.276472243	ath-miR396a-5p	730.4957811	691.1768887
ath-miR5026	1931.636666	1828.140614	ath-miR862-5p	12.29478581	12.21942118
ath-miR396a-5p	691.1768887	590.9983746	ath-miR172e-5p	8.455883908	7.263722407
ath-miR862-5p	12.21942118	8.642405897	ath-miR837-5p	1.945200255	1.698257592
ath-miR172e-5p	7.263722407	8.40294238	ath-miR398b-5p	6245.916192	8524.547583
ath-miR837-5p	1.698257592	1.887990221	ath-miR8175	281.2983609	646.2331839
ath-miR398b-5p	8524.547583	7790.115776	ath-miR8174	16.0564398	5.364887294
ath-miR8175	646.2331839	513.8692878	ath-miR8173	25.57364615	33.81001088
ath-miR8174	5.364887294	5.849997893	ath-miR408-3p	1563.913077	1166.137728
ath-miR8173	33.81001088	33.50489694	ath-miR8172	0.778080102	2.612906648
ath-miR408-3p	1166.137728	1628.343599	ath-miR8171	33.48599419	68.02921028
ath-miR8172	2.612906648	3.084641292	ath-miR169a-5p	53.92487177	61.90744948
ath-miR8171	68.02921028	56.02885517	ath-miR5012	20.31013106	6.924070888
ath-miR169a-5p	61.90744948	53.58278326	ath-miR160c-3p	280.6204775	367.1202299
ath-miR5012	6.924070888	6.514952159	ath-miR8168	5.006022056	0.679303037
ath-miR160c-3p	367.1202299	308.3044868	ath-miR8166	55.58682968	69.56165885
ath-miR8168	0.679303037	5.158658743	ath-miR8165	11.56820432	11.77546417
ath-miR8166	69.56165885	50.52386033	ath-miR399b	19.55780026	16.49581692
ath-miR8165	11.77546417	4.972631513	ath-miR169g-3p	35.61143957	108.527946
ath-miR399b	16.49581692	9.625978335	ath-miR399a	0.778080102	6.009421831
ath-miR169g-3p	108.527946	58.34166962	ath-miR5995b	0.778080102	2.00314061
ath-miR399a	6.009421831	1.701962991	ath-miR396a-3p	2870.553058	4671.177745
ath-miR5995b	2.00314061	1.196651071	ath-miR319c	168.4912934	265.2649418
ath-miR396a-3p	4671.177745	3364.322752	ath-miR319b	715.5896585	972.7638049
ath-miR319c	265.2649418	244.9354164	ath-miR862-3p	0.778080102	1.933603612
ath-miR319b	972.7638049	848.0328926	ath-miR172e-3p	0.389040051	0.339651518
ath-miR862-3p	1.933603612	2.393302141	ath-miR319a	1117.081247	1325.583903
ath-miR172e-3p	0.339651518	0.771160323	ath-miR837-3p	1.945200255	3.396515183
ath-miR319a	1325.583903	1243.101109	ath-miR173-5p	96.1214424	143.1054985
ath-miR837-3p	3.396515183	6.780800561	ath-miR168a-5p	1236.155507	1151.254565
ath-miR173-5p	143.1054985	170.7173316	ath-miR398b-3p	487.088801	412.1333421
ath-miR168a-5p	1151.254565	1042.285473	ath-miR399c-5p	27.57314551	15.31105335
ath-miR398b-3p	412.1333421	439.7737962	ath-miR169a-3p	72.18680536	72.43664654
ath-miR399c-5p	15.31105335	29.25065598	ath-miR5635d	12.32053511	10.35535457
ath-miR169a-3p	72.43664654	67.06389669	ath-miR5635c	6.147392906	6.279536351
ath-miR5635d	10.35535457	13.53521624	ath-miR5635b	6.147392906	6.279536351
ath-miR5635c	6.279536351	4.972631513	ath-miR5635a	4.980272753	1.968372111
ath-miR5635b	6.279536351	4.972631513	ath-miR3440b-3p	2.282741699	4.241627241
ath-miR5635a	1.968372111	2.659150544	ath-miR397b	40.85200586	65.44303866
ath-miR3440b-3p	4.241627241	6.648209619	ath-miR397a	367.1984194	135.6679336
ath-miR397b	65.44303866	51.26863577	ath-miR403-5p	34.18682638	36.86687455
ath-miR397a	135.6679336	147.7434973	ath-miR163	2206.033444	4012.797441
ath-miR403-5p	36.86687455	34.64877824	ath-miR396b-5p	2509.575466	2035.243812
ath-miR163	4012.797441	3064.676667	ath-miR863-5p	68.76549331	37.17979104
ath-miR396b-5p	2035.243812	2213.409489	ath-miR167a-5p	2773.057957	3610.556665
ath-miR863-5p	37.17979104	42.09453307	ath-miR173-3p	67.02348699	85.02245427
ath-miR167a-5p	3610.556665	3667.865686	ath-miR156f-5p	3512.709364	5306.64928
ath-miR173-3p	85.02245427	79.32264069	ath-miR398c-5p	6236.810975	8529.094094
ath-miR156f-5p	5306.64928	4538.225455	ath-miR168a-3p	173.7061104	177.5252754
ath-miR398c-5p	8529.094094	7790.939706	ath-miR169b-5p	29.10355641	17.854423
ath-miR168a-3p	177.5252754	155.6655131	ath-miR399c-3p	20.33588037	18.12453752
ath-miR169b-5p	17.854423	17.47083902	ath-miR393a-5p	733.9563002	237.4536847
ath-miR399c-3p	18.12453752	8.934639185	ath-miR395f	15.84764488	13.98721577
ath-miR393a-5p	237.4536847	319.1251167	ath-miR395e	3.838901903	4.650815758
ath-miR395f	13.98721577	19.1991869	ath-miR395d	3.838901903	3.971512722
ath-miR395e	4.650815758	3.696159269	ath-miR395c	18.51942663	16.60012242
ath-miR395d	3.971512722	3.696159269	ath-miR395b	18.51942663	16.60012242
ath-miR395c	16.60012242	20.82132872	ath-miR395a	3.838901903	3.971512722
ath-miR395b	16.60012242	20.82132872	ath-miR8121	34.68166414	12.6981467
ath-miR395a	3.971512722	3.696159269	ath-miR403-3p	6713.008987	6245.403015
ath-miR8121	12.6981467	10.31731749	ath-miR166a-5p	1591.530409	1649.554957
ath-miR403-3p	6245.403015	6495.247108	ath-miR396b-3p	546.9500151	662.4562517
ath-miR166a-5p	1649.554957	1639.203669	ath-miR158a-5p	174.7814353	64.32781208
ath-miR396b-3p	662.4562517	578.4697831	ath-miR863-3p	3.838901903	6.549650871
ath-miR158a-5p	64.32781208	59.72501444	ath-miR161.2	3428.864891	2444.756213
ath-miR863-3p	6.549650871	7.046648964	ath-miR167a-3p	441.768708	563.460014
ath-miR161.2	2444.756213	2279.770716	ath-miR156f-3p	8.430134605	3.631861203
ath-miR167a-3p	563.460014	659.8954922	ath-miR168b-5p	391.0362051	453.2605454
ath-miR156f-3p	3.631861203	3.855801615	ath-miR398c-3p	487.088801	412.1333421
ath-miR168b-5p	453.2605454	382.0957478	ath-miR394a	173.9865522	170.7322451
ath-miR398c-3p	412.1333421	439.7737962	ath-miR169b-3p	6.173142209	8.213139963
ath-miR394a	170.7322451	224.6741689	ath-miR8167f	80.9029828	65.58211265
ath-miR169b-3p	8.213139963	11.91307442	ath-miR8167e	80.9029828	65.58211265
ath-miR8167f	65.58211265	69.11086274	ath-miR8167d	80.9029828	65.58211265
ath-miR8167e	65.58211265	69.11086274	ath-miR8167c	80.9029828	65.58211265
ath-miR8167d	65.58211265	69.11086274	ath-miR8167b	80.9029828	65.58211265
ath-miR8167c	65.58211265	69.11086274	ath-miR781b	9.545756151	6.279536351
ath-miR8167b	65.58211265	69.11086274	ath-miR8167a	80.9029828	65.58211265
ath-miR781b	6.279536351	2.659150544	ath-miR781a	9.545756151	6.279536351
ath-miR8167a	65.58211265	69.11086274	ath-miR822-5p	1425.979047	1812.242636
ath-miR781a	6.279536351	2.659150544	ath-miR393a-3p	35.95178151	11.74872914
ath-miR822-5p	1812.242636	2017.052507	ath-miR394b-5p	172.041352	167.4748039
ath-miR393a-3p	11.74872914	30.47435846	ath-miR165a-5p	4715.102402	3174.091551
ath-miR394b-5p	167.4748039	224.0890358	ath-miR157a-5p	16636.67893	24107.1781
ath-miR165a-5p	3174.091551	4686.400166	ath-miR166a-3p	19026.56177	14328.73218
ath-miR157a-5p	24107.1781	17602.06083	ath-miR5665	2.334240306	1.018954555
ath-miR166a-3p	14328.73218	19586.25202	ath-miR158a-3p	372189.4679	184557.1448
ath-miR5665	1.018954555	2.659150544	ath-miR864-5p	14.86357035	7.838719945
ath-miR158a-3p	184557.1448	203105.8327	ath-miR839-5p	18.62242384	15.88605089
ath-miR864-5p	7.838719945	3.855801615	ath-miR1888b	2.282741699	0
ath-miR839-5p	15.88605089	15.98195467	ath-miR1888a	11.05041775	3.056863665
ath-miR1888b	0	2.393302141	ath-miR159b-5p	26.04273461	10.18151207
ath-miR1888a	3.056863665	4.148034902	ath-miR168b-3p	125.5395915	121.6192143
ath-miR159b-5p	10.18151207	12.73700451	ath-miR4221	0.778080102	0
ath-miR168b-3p	121.6192143	117.0296753	ath-miR391-5p	137.3109897	313.6719345
ath-miR4221	0	1.542320646	ath-miR5659	3.838901903	1.018954555
ath-miR391-5p	313.6719345	231.3474304	ath-miR5657	4.980272753	2.377560628
ath-miR5659	1.018954555	4.547140765	ath-miR5656	1.556160204	4.920930278
ath-miR5657	2.377560628	1.967811394	ath-miR822-3p	14.34298328	17.72338248
ath-miR822-3p	17.72338248	29.35752856	ath-miR5652	11.56820432	25.69314294
ath-miR5652	25.69314294	25.90016629	ath-miR5651	9.23396401	15.13721085
ath-miR5651	15.13721085	13.37557389	ath-miR393b-5p	733.9563002	237.4536847
ath-miR393b-5p	237.4536847	319.1251167	ath-miR394b-3p	3.838901903	2.308023629
ath-miR394b-3p	2.308023629	5.743791836	ath-miR156a-5p	3507.314302	5302.947881
ath-miR156a-5p	5302.947881	4537.879785	ath-miR165a-3p	15090.48371	9545.32932
ath-miR860	9.336400016	3.084641292	ath-miR157a-3p	456.1430416	216.2829516
ath-miR165a-3p	9545.32932	16683.60911	ath-miR829-5p	0	1.698257592
ath-miR157a-3p	216.2829516	309.2893923	ath-miR166b-5p	1591.530409	1649.554957
ath-miR829-5p	1.698257592	1.542320646	ath-miR5644	45.15719572	54.13826653
ath-miR166b-5p	1649.554957	1639.203669	ath-miR5640	43.06030014	9.405937014
ath-miR5644	54.13826653	47.19975553	ath-miR159b-3p	4307.766267	3865.552375
ath-miR5640	9.405937014	9.865441853	ath-miR2112-5p	23.78574222	13.81337328
ath-miR159b-3p	3865.552375	3943.681899	ath-miR857	16.0049412	12.7676837
ath-miR2112-5p	13.81337328	20.87409849	ath-miR390a-5p	2897.733895	2222.150794
ath-miR857	12.7676837	5.158658743	ath-miR853	4.980272753	0
ath-miR390a-5p	2222.150794	3197.72907	ath-miR852	3.89040051	8.691865478
ath-miR853	0	1.037008725	ath-miR850	406.949351	458.055834
ath-miR852	8.691865478	4.307677247	ath-miR391-3p	1452.250725	2552.591424
ath-miR850	458.055834	425.1421363	ath-miR5637	25.08160889	11.98407516
ath-miR391-3p	2552.591424	2020.130093	ath-miR5996	7.314513059	21.95697624
ath-miR5637	11.98407516	9.865441853	ath-miR171a-5p	16.18518632	10.28581757
ath-miR5996	21.95697624	8.9082543	ath-miR5595a	0.778080102	2.00314061
ath-miR171a-5p	10.28581757	15.79526092	ath-miR5634	8.844923959	17.715349
ath-miR5595a	2.00314061	1.196651071	ath-miR393b-3p	639.6602578	336.2547438
ath-miR5634	17.715349	9.546157162	ath-miR773a	54.1851653	51.59489688
ath-miR393b-3p	336.2547438	370.2888794	ath-miR848	31.84978558	57.23793217
ath-miR773a	51.59489688	49.19461832	ath-miR847	35.25094932	16.53058542
ath-miR848	57.23793217	69.13791414	ath-miR843	124.7928617	91.18965165
ath-miR847	16.53058542	24.80972128	ath-miR156a-3p	48.47831105	33.9758199
ath-miR843	91.18965165	75.86794449	ath-miR842	21.29420559	14.29209879
ath-miR156a-3p	33.9758199	52.3056445			
ath-miR842	14.29209879	25.76757535			
